# Prevalence and characteristics of medical and rehabilitation utilization among Canadians with arthritis from 2001 to 2018: a cross-sectional population-based study

**DOI:** 10.1186/s12913-025-13471-5

**Published:** 2025-10-02

**Authors:** Sheilah Hogg-Johnson, Dan Wang, Jessica J. Wong, Silvano A. Mior, Pierre Côté

**Affiliations:** 1 Department of Research and Innovation, Canadian Memorial Chiropractic College, 6100 Leslie Street, Toronto, Ontario M2H 3J1 Canada; 2 Institute for Disability and Rehabilitation Research, Ontario Tech University, 2000 Simcoe Street North, Oshawa, Ontario L1H 7K4 Canada; 3 Dalla Lana School of Public Health, University of Toronto, 155 College Street, 6th floor, Toronto, Ontario M5T 3M7 Canada; 4 Institute of Health Policy, Management and Evaluation, University of Toronto, 155 College Street, 4th Floor, Toronto, Ontario M5T 3M6 Canada; 5 Graduate Studies, Canadian Memorial Chiropractic College, 6100 Leslie Street, Toronto, Ontario M2H 3J1 Canada; 6 Faculty of Health Sciences, Ontario Tech University, 2000 Simcoe Street North, Oshawa, Ontario L1G 0C5 Canada; 7 School of Physical Therapy, Faculty of Health Sciences, Western University, 1201 Western Road, London, Ontario N6G 1H1 Canada; 8 Department of Epidemiology and Biostatistics, Schulich School of Medicine & Dentistry, Western University, 1151 Richmond Street, London, Ontario N6A 5C1 Canada

**Keywords:** Arthritis, Health care utilization, Prevalence, Population-based study

## Abstract

**Background:**

Arthritis covers a range of chronic diseases presenting as joint pain and inflammation with prevalence of 20% in Canadians. Treatment guidelines for arthritis depend upon the type of arthritis but most include recommendations for rehabilitation interventions designed to optimize functioning and reduce disability. We set out to estimate the prevalence of healthcare utilization with different providers and to explore factors associated with utilization of different providers among Canadians with arthritis.

**Methods:**

This population-based study used Canadian Community Health Survey data (2001–2018) restricted to respondents with arthritis (≥12 years). We used self-reported consultation with healthcare providers (medical doctor, chiropractor, physiotherapist, nurse, psychologist) (2001–2010), and self-reported regular healthcare provider (2015–2018). We calculated the 12-month prevalence of utilization with providers, and used modified Poisson regression to assess predisposing (e.g. age, sex, education), enabling (e.g. income, province) and need (e.g. self-percieved health) factors associated with utilization of providers.

**Results:**

From 2001–2010 and 2015–2018, respectively, prevalence of utilization of medical doctors was 92.0% (95%CI: 91.7–92.2%) and 91.0% (95%CI: 90.5–91.5%); chiropractors 13.1% (12.8–13.4%) and 9.6% (9.1–10.1%); physiotherapists 14.5% (14.1–14.8%) and 9.4% (8.9–9.9%); nurses 14.2% (13.9–14.5%) and 7.5% (7.2–7.9%); psychologists 3.0% (2.8–3.1%) and 3.9% (3.5–4.2%). Females were more likely to see any provider. Users of chiropractic care were less likely to be smokers and more physically active with greater utilization in the western provinces than in the east. Those with poorer self-perceived health were more likely to see physiotherapists, nurses and psychologists. Consultation with a nurse (2001–2010) was more likely in the northern territories, while regular care from a nurse (2015–2018) was more likely in older age groups.

**Conclusions:**

Canadians with arthritis were most likely to see medical doctors. Characteristics of healthcare utilizers varied by provider type. Geographical variation in utilization of chiropractors and physiotherapists likely related to differences by province and over time in what provincial health insurance covered while geographical variation in utilization of nurses was likely related to the lack of availability of medical doctors. Findings inform the need to strengthen healthcare delivery for Canadians, perhaps providing better access to providers of rehabilitation interventions.

## Introduction

Arthritis is a term that covers a range of chronic diseases presenting as joint pain and inflammation. There are five major types: osteoarthritis (OA), rheumatoid arthritis (RA), psoriatic arthritis (PA), ankylosing spondylitis (AS) and gout [1] with osteoarthritis being the most common. Based on global burden of disease estimates, OA accounts for approximately 85% of all prevalent arthritis [2–5]. Common symptoms include pain, stiffness, swelling and reduced range of motion, and there is often considerable associated functional limitations and disability [6]. The Arthritis Society of Canada reports that 20% of the Canadian population has arthritis with that percentage expected to grow to 24% by 2040 [7], while the Center for Disease Control (CDC) reports 22.7% of adults in the United States (US) have some form of arthritis [8].

Appropriate evidence-based treatment for arthritis is dependent upon the type of arthritis and treatment guidelines for different types of arthritis have been developed. For osteoarthritis, which affects more people than all other types of arthritis combined and accounts for 10 times as many cases as rheumatoid arthritis [9], there are surgical options via total joint arthropathy e.g., for hip and knee arthritis [10] and non-surgical options available and recommended [11]. Guideline recommendations for OA include pharmacological options such as topical and oral non-steroidal anti-inflammatories (NSAIDs) but also rehabilitation interventions such as exercise, manual therapy and weight management [11]. While pharmacological management of RA is a first line of treatment approach [12], guidelines also include recommendations for the inclusion of non-pharmacological approaches such as exercises (fitness, joint mobility, muscle strength) and pain-relieving modalities such as transcutaneous electrical nerve stimulation (TENS) [13]. Similarly, for PA and AS, guidelines focus on pharmacologic management, but also include recommendations for exercise and physiotherapy [14–19].

According to the World Health Organization (WHO), rehabilitation is “a set of interventions designed to optimize functioning and reduce disability in individuals with health conditions in interaction with their environment” which includes exercise, manual therapy and cognitive behavioral therapy [20]. In a recent scoping review, Kamenov et al. concluded there are substantial unmet rehabilitation needs globally [21], with “numerous barriers to accessing services”. Ronksley and colleagues [22] identified unmet health care needs among Canadians with chronic conditions, and noted an increased odds of unmet needs among those with arthritis. The WHO Rehabilitation 2030 Initiative draws attention to the global unmet need for rehabilitation and the need to strengthen health systems to improve access to rehabilitation [20]. According to the WHO, there are 344 million people living with OA that could benefit from rehabilitation [23].

In the Canadian context, the Canada Health Act [24] requires universal coverage of insured health services which include hospital services and physician services, with insurance plans overseen at the provincial and territorial levels. Data suggest that 71% of health expenditures in Canada are paid for by public sector funds [25]. However, there is currently limited coverage for rehabilitation services in the provincial schemes, rather such services are generally paid for by provincial workers’ compensation schemes, health benefits provided through employment, automobile insurance or paid for by individuals out of pocket. Moreover, there is some variation in what is covered by provincial and territorial health insurance plans, and there has been variation in that coverage over time.

The Canadian Community Health (CCHS) surveys comprise a series of cross-sectional surveys conducted by Statistics Canada that can be used to study population level information about Canadians related to health determinants, health status and health system utilization [26]. They provide an opportunity to examine health system utilization among Canadian adults living with arthritis. To our knowledge, there have been no studies looking at healthcare utilization, including a range of providers, of adult Canadians with arthritis over time. We set out to: 1) estimate the prevalence of healthcare utilization with different providers (medical doctors, chiropractors, physiotherapists, psychologists, and nurses); and 2) explore factors associated with the utilization of different healthcare providers in Canadian adults self-identifying as living with arthritis. We are using a population health based approach to our analysis, and given the challenges facing the Canadian healthcare system, this approach is informative and useful to explore utilization in an historical context.

## Materials and methods

### Study design

We analyzed cross-sectional data collected in seven cycles of the CCHS from 2001 to 2010 (5 cycles) and 2015–2018 (2 cycles). The study is reported according to the Strengthening the Reporting of Observational Studies in Epidemiology (STROBE) Statement [27] (Appendix 1). The study protocol was reviewed and approved by the by the Research Ethics Board at Ontario Tech University (Reference# 15791–130103).

### Canadian community health survey

The CCHS is a cross-sectional survey of a representative sample of the Canadian population with data collected every two years from 2001 to 2007, after which survey data were collected annually [26]. It is a joint initiative of Canadian Institute for Health Information (CIHI), Statistics Canada and Health Canada and it collects information about the health status, health care utilization and health determinants of the Canadian population. It uses a multi-stage sampling strategy to obtain representative data from persons aged 12 and over living in private dwellings in the Canadian provinces and territories. For this study, data from the 5 cycles between 2001 and 2010 inclusive and from 2015 and 2018 inclusive were utilized as these cycles included questions necessary for the analysis presented here. In particular, for inclusion in this study, respondents had to have answered yes to the question:

*“[Now we are interested in “long-term conditions” which are expected to last or have already lasted 6 months or more and that have been diagnosed by a health professional]*

*Do you have arthritis (or rheumatism, e.g., osteoarthritis, rheumatoid arthritis, gout or any other type) excluding fibromyalgia?”*

Data from the Public Use Microdata File (PUMF) of the Canadian Community Health Survey were accessed via the Ontario Data Documentation, Extraction Service and Infrastructure Initiative (ODESI). Data from 2001 to 2010 were pooled together for one set of analyses and data from 2015 to 2018 were pooled together for the other set of analyses given wording of questions about health care utilization were different for these two periods of time.

### Outcome measures

#### Consultation with healthcare provider (2001–2010)

Outcomes for 2001–2010 CCHS cycles were consultations with medical doctors, nurses, chiropractors, physiotherapists or psychologists over the previous 12 months.

1. 2001-2005 cycles: *“Not counting when you were an overnight patient, in the past 12 months, how many times have you seen, or talked to a medical doctor, nurse, chiropractor, physiotherapist, or psychologist (about your physical, emotional or mental health)?” (≥1 consultation considered as yes to having consulted each provider)*
2. 2007 and 2009 cycles: *“Not counting when you were an overnight patient, in the past 12 months, have you seen, or talked to a medical doctor, nurse, chiropractor, physiotherapist, or psychologist (about your physical, emotional or mental health)?” (responding “yes”).*

In this study, consultation with a medical doctor includes with a family doctor or general practitioner or consultation with any other medical doctor or specialist such as surgeon, allergist, orthopedist, gynaecologist or psychiatrist. Note that respondents could choose more than one of these options in their responses.

#### Regular healthcare provider (2015– 2018)

The outcome for the 2015 and 2018 CCHS cycles was self-report of receiving regular healthcare from medical doctors, nurses, chiropractors, physiotherapists or psychologists.

This was based on three CCHS questions:

1. *“Do you have a regular healthcare provider? (one health professional that you regularly see or talk to when you need care or advice for your health)” (responding “yes”);*
2. *“Is that regular healthcare provider a …” with response options of ‘family doctor/general practitioner’, ‘medical specialist’, ‘nurse’, or ‘other’;*
3. *“Other than from the above regular healthcare provider, who else do you receive regular healthcare from (regular healthcare can also be considered as routine healthcare)?” with 10 response options that listed different healthcare providers.*

The outcome of receiving regular care from medical doctors (including specialists) included response options of “family doctor/general practitioner” or “medical specialist” from question #2; or “another family doctor/general practitioner” or “specialist doctor” from question #3. The outcome of regular care from chiropractors, physiotherapists or psychologists was based on selecting the response of “chiropractor”, “physiotherapist”, or “psychologist” respectively from question #3. The outcome of regular care from nurses was based on selecting the response of “nurse” from question #2 or “another nurse or nurse practitioner” from question #3. Note that respondents could choose more than one of these options in their responses.

Although these questions have not been assessed for validity or reliability, previous studies have used them to describe healthcare utilization in Canada [28–31].

### Covariates

Informed by literature [32, 33] we hypothesized that the following factors would be associated with utilization of healthcare providers, grouped as predisposing (demographic, social, and belief factors that predispose individuals to use or not use health services, irrespective of their health conditions or need), enabling (health policy, financing, and organizational resources that can facilitate or impede use of services such as the ability or inability to pay) and need (health conditions recognized by individuals and health care providers as requiring health services such as lifestyle risk factors and existing health conditions) based on the Anderson Health Care Utilization model [32, 34].

- Predisposing: age, sex, cultural/racial background, immigrant status, education, marital status.
- Enabling: province/territory of residence, income, working status.
- Need: body mass index (BMI), smoking status, drinking status, physical activity level, perceived general health.

The survey questions used for all covariates are provided in Appendix 2. In particular, household income is a derived variable with a slightly different derivation in the two earliest CCHS cycles. From 2005 on, it represents income quintiles, a relative measure of household income based on rank order of household income of all CCHS respondents applying population weights. Quintile 1 indicates household income in the bottom 20% of Canadians, whereas Quintile 5 represents household income in the top 20% of Canadians. We also included CCHS cycle in the models for years 2001–2010 to assess for differences in the prevalence of healthcare utilization over time.

### Statistical analysis

We constructed 12-month prevalence estimates of health care utilization using the number of respondents with arthritis consulting with (or receiving regular care from) a provider as the numerator and the total number of respondents with arthritis in the denominator.

We used modified Poisson regression to model the associations between predisposing, enabling and need covariates and outcomes of health care utilization for each provider type to estimate crude and adjusted Prevalence Ratios (PR) with 95% confidence intervals (CI). As there were no 12–19 year old Canadians with arthritis in the pooled sample from CCHS 2015–2018, we excluded this age group from both time periods for the Poisson regression models. Participants with missing data on covariates were excluded from regression analyses ( < 10%).

All analyses incorporated the CCHS survey weights provided by Statistics Canada to generate population estimates and bootstrap weights were applied using balanced repeated replications for CCHS 2015–2018. We used a pooled approach to combine data across CCHS cycles, which increases sample size and statistical power [35]. Weights were adjusted for the pooled approach by dividing the original weights by the number of survey cycles [35]. The analysis for this study was generated using SAS software v9.4. (Copyright © 2012–2018, SAS Institute Inc., Cary, NC, USA. SAS and all other SAS Institute Inc. product or service names are registered trademarks or trademarks of SAS Institute Inc., Cary, NC, USA.)

## Results

### Sample characteristics (Figs. 1 & 2 flow of participants)

**Fig. 1:**
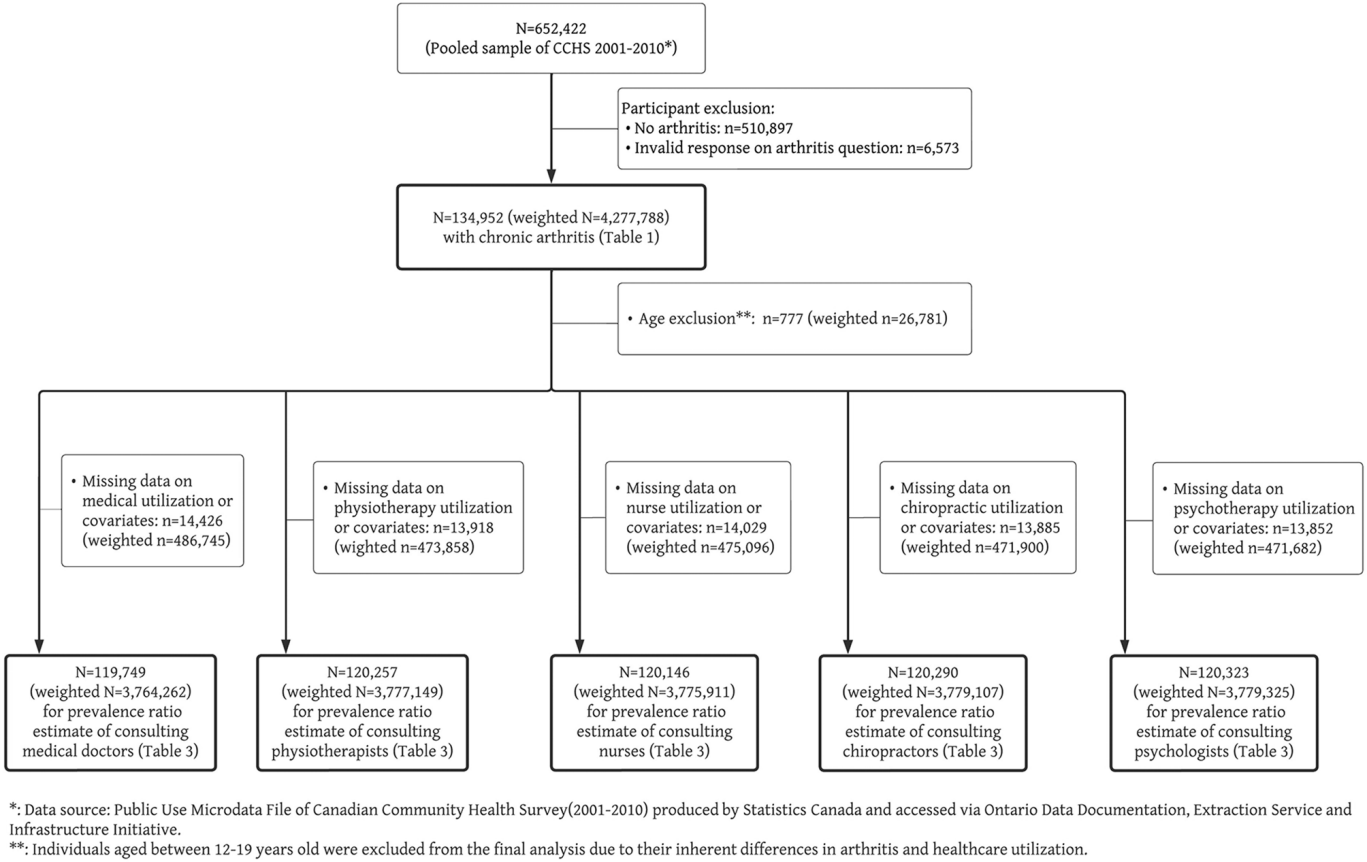
Flow of participants used in analysis from CCHS 2001–2010

**Fig. 2:**
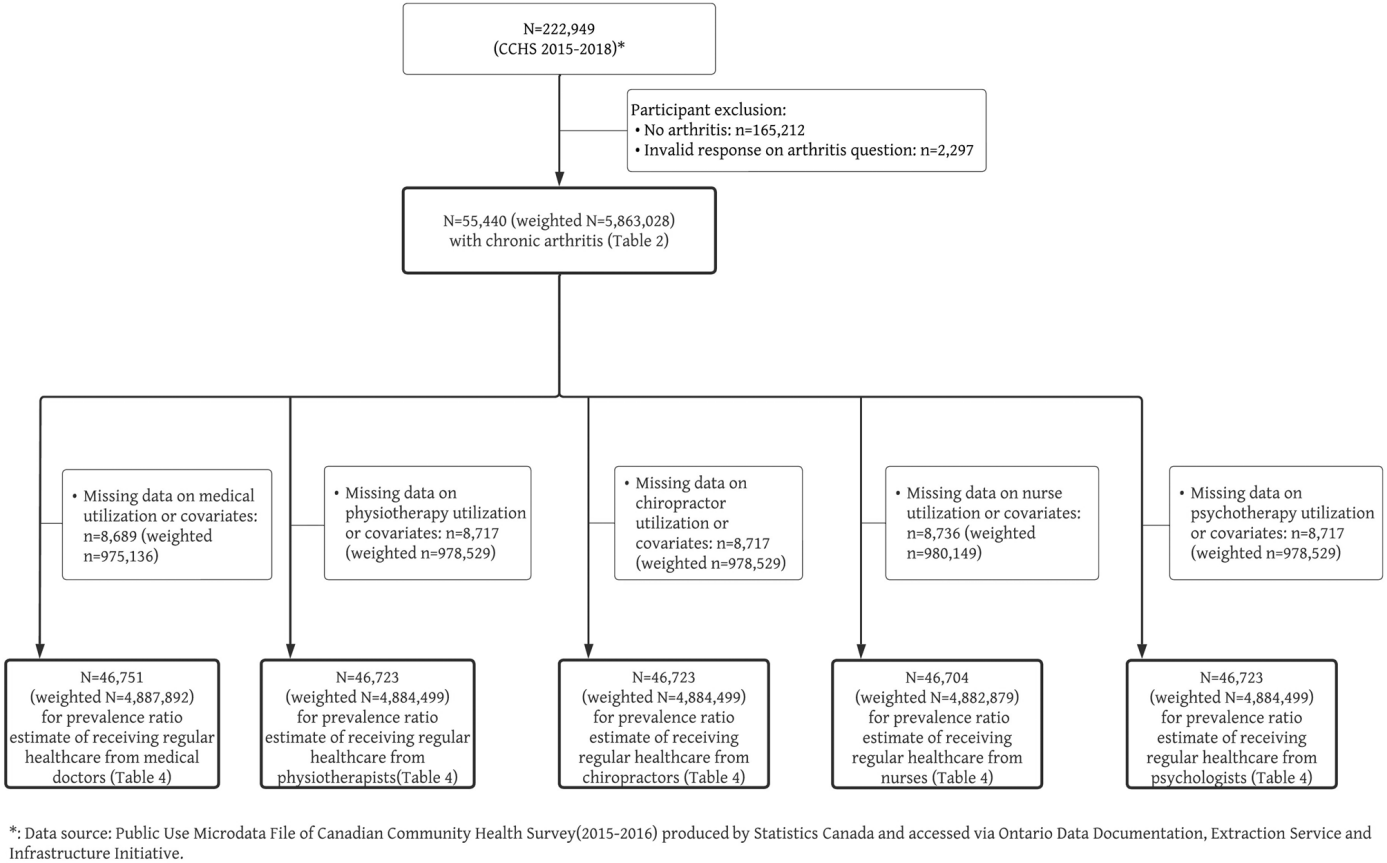
Flow of participants used in analysis from CCHS 2015–2018

A total of 652,422 Canadians participated in the CCHS between 2001 and 2010 (Fig. 1) and a total of 222,949 participated between 2015 and 2018 (Fig. 2). After applying inclusion/exclusion criteria (reported having arthritis), the 2001–2010 study sample included 134,952 respondents (weighted to population 4,277,788), with weighted estimated prevalence of arthritis of 15.7% (95%CI: 15.6–15.8). The 2015–2018 sample included 55,440 respondents (weighted to population 5,863,028) with weighted estimated prevalence of arthritis of 19.0% (95%CI: 18.7–19.2).

### Canadians with arthritis (Tables 1 and 2)

#### 2001–2010: Consultation with healthcare provider (Table 1)

**Table 1:** Characteristics of participants with arthritis and those who consulted health professionals: pooled analysis of CCHS 2001–2010

		Consultations with health care professionals among Canadians with chronic arthritis				
Characteristics	Canadians with chronic arthritis	Medical doctor (incl. specialists)	Physiotherapist	Nurse	Chiropractor	Psychologist
	N (%)	n (%)	n (%)	n (%)	n (%)	n (%)
**Weighted sample**	4277788 (100.0%)	3933694 (100.0%)	618802 (100.0%)	609239 (100.0%)	558545 (100.0%)	127406 (100.0%)
**% Prevalence Consulting HCP (95%CI)**		92.0 (91.7–92.2)	14.5 (14.1–14.8)	14.2 (13.9–14.5)	13.1 (12.8–13.4)	3.0 (2.8–3.1)
**Age group (years)**						
12–19	26781 (0.6%)	22937 (0.6%)	5582 (0.9%)	6007 (1.0%)	4060 (0.7%)	2506 (2.0%)
20–34	207314 (4.8%)	182643 (4.6%)	32602 (5.3%)	41577 (6.8%)	35075 (6.3%)	13102 (10.3%)
35–49	774326 (18.1%)	698720 (17.8%)	137184 (22.2%)	114901 (18.9%)	132291 (23.7%)	44485 (34.9%)
50–64	1505563 (35.2%)	1382349 (35.1%)	235036 (38.0%)	191927 (31.5%)	219976 (39.4%)	50535 (39.7%)
65–79	1316816 (30.8%)	1228835 (31.2%)	160824 (26.0%)	166235 (27.3%)	136709 (24.5%)	13193 (10.4%)
80+	446988 (10.4%)	418210 (10.6%)	47574 (7.7%)	88591 (14.5%)	30434 (5.4%)	3586 (2.8%)
**Sex**						
Male	1612920 (37.7%)	1452778 (36.9%)	221411 (35.8%)	211975 (34.8%)	212544 (38.1%)	44734 (35.1%)
Female	2664868 (62.3%)	2480915 (63.1%)	397391 (64.2%)	397264 (65.2%)	346001 (61.9%)	82672 (64.9%)
**Province of residence**						
Newfoundland and Labrador	89392 (2.1%)	83770 (2.1%)	8860 (1.4%)	12042 (2.0%)	4965 (0.9%)	1648 (1.3%)
Prince Edward Island	22671 (0.5%)	21234 (0.5%)	3071 (0.5%)	3179 (0.5%)	1130 (0.2%)	393 (0.3%)
Nova Scotia	184901 (4.3%)	173064 (4.4%)	25599 (4.1%)	21168 (3.5%)	10118 (1.8%)	5943 (4.7%)
New Brunswick	123804 (2.9%)	114688 (2.9%)	14855 (2.4%)	18997 (3.1%)	8712 (1.6%)	3548 (2.8%)
Quebec	791609 (18.5%)	715612 (18.2%)	90176 (14.6%)	154462 (25.4%)	69292 (12.4%)	33051 (25.9%)
Ontario	1782720 (41.7%)	1647588 (41.9%)	261569 (42.3%)	234382 (38.5%)	234065 (41.9%)	44585 (35.0%)
Manitoba	163959 (3.8%)	149217 (3.8%)	26704 (4.3%)	19697 (3.2%)	31904 (5.7%)	3870 (3.0%)
Saskatchewan	147301 (3.4%)	135156 (3.4%)	19659 (3.2%)	20560 (3.4%)	26776 (4.8%)	2874 (2.3%)
Alberta	413747 (9.7%)	377463 (9.6%)	67395 (10.9%)	60089 (9.9%)	85367 (15.3%)	15919 (12.5%)
British Columbia	548744 (12.8%)	508129 (12.9%)	99421 (16.1%)	61473 (10.1%)	85333 (15.3%)	15245 (12.0%)
Yukon/Northwest Territories/Nunavut (territories)	8940 (0.2%)	7773 (0.2%)	1491 (0.2%)	3188 (0.5%)	882 (0.2%)	329 (0.3%)
**Cultural/racial background**						
White	3717528 (86.9%)	3425598 (87.1%)	531095 (85.8%)	545662 (89.6%)	497961 (89.2%)	109445 (85.9%)
Non-white	432744 (10.1%)	395629 (10.1%)	71037 (11.5%)	46707 (7.7%)	47860 (8.6%)	14545 (11.4%)
	127516 (3.0%)	112466 (2.9%)	16671 (2.7%)	16869 (2.8%)	12724 (2.3%)	3417 (2.7%)
**Immigrant status**						
Non-immigrant	3258304 (76.2%)	2989499 (76.0%)	470029 (76.0%)	501998 (82.4%)	453256 (81.1%)	105105 (82.5%)
Immigrant(0–9 years)	64238 (1.5%)	59570 (1.5%)	9480 (1.5%)	4001 (0.7%)	7246 (1.3%)	1853 (1.5%)
Immigrant(10+ years)	844084 (19.7%)	787263 (20.0%)	125868 (20.3%)	88905 (14.6%)	87599 (15.7%)	17747 (13.9%)
	111162 (2.6%)	97362 (2.5%)	13425 (2.2%)	14335 (2.4%)	10444 (1.9%)	2702 (2.1%)
**Highest level of education - respondents**						
< secondary	1320035 (30.9%)	1204620 (30.6%)	129037 (20.9%)	185856 (30.5%)	126693 (22.7%)	24827 (19.5%)
Secondary grad	698075 (16.3%)	641682 (16.3%)	96807 (15.6%)	85383 (14.0%)	91226 (16.3%)	17808 (14.0%)
some post-sec edu	263373 (6.2%)	243423 (6.2%)	38475 (6.2%)	42834 (7.0%)	39759 (7.1%)	10269 (8.1%)
Post-sec grad/university degree	1871077 (43.7%)	1734365 (44.1%)	338746 (54.7%)	279854 (45.9%)	289257 (51.8%)	71636 (56.2%)
.	125229 (2.9%)	109603 (2.8%)	15738 (2.5%)	15312 (2.5%)	11610 (2.1%)	2867 (2.3%)
**Total household income**						
1st quintile	650782 (15.2%)	596393 (15.2%)	73385 (11.9%)	114349 (18.8%)	53814 (9.6%)	24945 (19.6%)
2nd quintile	639323 (14.9%)	587339 (14.9%)	80363 (13.0%)	92935 (15.3%)	73254 (13.1%)	19072 (15.0%)
3rd quintile	775445 (18.1%)	717741 (18.2%)	103052 (16.7%)	117491 (19.3%)	103032 (18.4%)	21000 (16.5%)
4th quintile	837909 (19.6%)	773441 (19.7%)	138770 (22.4%)	109767 (18.0%)	131836 (23.6%)	25677 (20.2%)
5th quintile	660643 (15.4%)	614339 (15.6%)	131668 (21.3%)	84002 (13.8%)	115995 (20.8%)	21995 (17.3%)
NA/NS*	713687 (16.7%)	644441 (16.4%)	91563 (14.8%)	90694 (14.9%)	80615 (14.4%)	14717 (11.6%)
**Working status last week**						
Working	1489718 (34.8%)	1340080 (34.1%)	240563 (38.9%)	178940 (29.4%)	265546 (47.5%)	49698 (39.0%)
Absent	149482 (3.5%)	138288 (3.5%)	36083 (5.8%)	28063 (4.6%)	26403 (4.7%)	8767 (6.9%)
No job	1468157 (34.3%)	1357879 (34.5%)	194188 (31.4%)	192967 (31.7%)	168231 (30.1%)	43778 (34.4%)
Unable/permanent	256623 (6.0%)	245250 (6.2%)	44552 (7.2%)	57613 (9.5%)	24652 (4.4%)	16197 (12.7%)
NA(age < 15 or > 75)	837951 (19.6%)	784987 (20.0%)	93720 (15.1%)	143442 (23.5%)	65567 (11.7%)	7089 (5.6%)
.	75857 (1.8%)	67208 (1.7%)	9697 (1.6%)	8214 (1.3%)	8145 (1.5%)	1876 (1.5%)
**Marital status**						
Married	2545326 (59.5%)	2356148 (59.9%)	384627 (62.2%)	321550 (52.8%)	356623 (63.8%)	56926 (44.7%)
Common-law	256817 (6.0%)	228769 (5.8%)	39409 (6.4%)	39658 (6.5%)	37486 (6.7%)	13657 (10.7%)
Widowed/Divorced/Separated	1068106 (25.0%)	987100 (25.1%)	135527 (21.9%)	177737 (29.2%)	114628 (20.5%)	32732 (25.7%)
Single	398766 (9.3%)	353676 (9.0%)	57683 (9.3%)	69064 (11.3%)	48685 (8.7%)	23593 (18.5%)
.	8774 (0.2%)	8001 (0.2%)	1556 (0.3%)	1230 (0.2%)	1123 (0.2%)	499 (0.4%)
**Type of smoker**						
Daily	787888 (18.4%)	698671 (17.8%)	96348 (15.6%)	109496 (18.0%)	89330 (16.0%)	34471 (27.1%)
Occasional	134197 (3.1%)	120312 (3.1%)	18745 (3.0%)	20251 (3.3%)	18867 (3.4%)	7620 (6.0%)
Not at all	3338836 (78.1%)	3100009 (78.8%)	501473 (81.0%)	477762 (78.4%)	447983 (80.2%)	84911 (66.6%)
.	16867 (0.4%)	14702 (0.4%)	2237 (0.4%)	1730 (0.3%)	2364 (0.4%)	404 (0.3%)
**Type of drinker**						
Regular	2198474 (51.4%)	2016717 (51.3%)	349193 (56.4%)	283195 (46.5%)	327229 (58.6%)	68245 (53.6%)
Occasional	850763 (19.9%)	789498 (20.1%)	114830 (18.6%)	135723 (22.3%)	108369 (19.4%)	25081 (19.7%)
Did not drink	1155437 (27.0%)	1063830 (27.0%)	146238 (23.6%)	181513 (29.8%)	115444 (20.7%)	32303 (25.4%)
.	73115 (1.7%)	63649 (1.6%)	8541 (1.4%)	8807 (1.4%)	7502 (1.3%)	1777 (1.4%)
**Physical activity**						
Active	759682 (17.8%)	686683 (17.5%)	125379 (20.3%)	97067 (15.9%)	117176 (21.0%)	22564 (17.7%)
Moderate active	933294 (21.8%)	861117 (21.9%)	145112 (23.5%)	125031 (20.5%)	136883 (24.5%)	27221 (21.4%)
Inactive	2404117 (56.2%)	2220358 (56.4%)	325697 (52.6%)	352194 (57.8%)	291131 (52.1%)	72425 (56.8%)
	180695 (4.2%)	165537 (4.2%)	22615 (3.7%)	34947 (5.7%)	13355 (2.4%)	5196 (4.1%)
**BMI classification - International standard**						
Underweight	87513 (2.0%)	79888 (2.0%)	11603 (1.9%)	17165 (2.8%)	9688 (1.7%)	3985 (3.1%)
Normal weight	1337983 (31.3%)	1217985 (31.0%)	193501 (31.3%)	179819 (29.5%)	174431 (31.2%)	44655 (35.0%)
Overweight (incl. obese)	2340426 (54.7%)	2161450 (54.9%)	349371 (56.5%)	332237 (54.5%)	324272 (58.1%)	68967 (54.1%)
NA (age < 18 or pregnant)**	335786 (7.8%)	315732 (8.0%)	41252 (6.7%)	50980 (8.4%)	34232 (6.1%)	5137 (4.0%)
.	176080 (4.1%)	158638 (4.0%)	23074 (3.7%)	29038 (4.8%)	15922 (2.9%)	4662 (3.7%)
**Perceived general health**						
Poor	389161 (9.1%)	372016 (9.5%)	68453 (11.1%)	98656 (16.2%)	37980 (6.8%)	22462 (17.6%)
Fair	897477 (21.0%)	843848 (21.5%)	136518 (22.1%)	162521 (26.7%)	103266 (18.5%)	33611 (26.4%)
Good	1512242 (35.4%)	1393765 (35.4%)	214084 (34.6%)	196728 (32.3%)	202640 (36.3%)	40001 (31.4%)
Very good	1101559 (25.8%)	996322 (25.3%)	154243 (24.9%)	117464 (19.3%)	161030 (28.8%)	24015 (18.8%)
Excellent	370601 (8.7%)	322112 (8.2%)	44701 (7.2%)	32976 (5.4%)	52969 (9.5%)	7152 (5.6%)
.	6748 (0.2%)	5631 (0.1%)	803 (0.1%)	894 (0.1%)	661 (0.1%)	166 (0.1%)
**Survey cycle**						
CCHS 2001	786095 (18.4%)	730039 (18.6%)	109721 (17.7%)	103748 (17.0%)	105961 (19.0%)	22648 (17.8%)
CCHS 2003	891046 (20.8%)	813383 (20.7%)	119716 (19.3%)	115666 (19.0%)	111540 (20.0%)	26336 (20.7%)
CCHS 2005	888370 (20.8%)	810828 (20.6%)	122684 (19.8%)	123174 (20.2%)	117095 (21.0%)	23654 (18.6%)
CCHS 2007	847449 (19.8%)	777873 (19.8%)	125318 (20.3%)	127106 (20.9%)	110711 (19.8%)	25601 (20.1%)
CCHS 2009	864829 (20.2%)	801571 (20.4%)	141364 (22.8%)	139544 (22.9%)	113237 (20.3%)	29168 (22.9%)

1. Data source: Public Use Microdata File of Canadian Community Health Survey produced by Statistics Canada and accessed via Ontario Data Documentation, Extraction Service and Infrastructure Initiative. 2. Five cycles from Canadian Community Health Survey 2001–2010 were pooled to obtain a single sample representing an average population over these ten years; Original sampling weights within each cycle were rescaled by dividing 5 (the number of survey cycles) to obtain a new weight in the newly pooled average population. All statistics are based on the rescaled sampling weights. The results or view expressed are those of the authors and are not those of Statistics Canada. *: NA = not applicable according to population exclusions. NS = not stated or responses without enough information for classification. For Total Household Income, to capture the large number of participants without sufficient information on income, Not Stated was merged with Not Applicable and treated as one category together. **: BMI = Weight (Kg)/Squared height (Meters), weight and height are self-reported. The BMI categories are adopted from a body weight classification system recommended by Health Canada and the World Health Organization (WHO) which has been widely used internationally. In CCHS 2001, NA: age < 20 or age > 64 or pregnant; In CCHS 2004, NA: age < 20 or pregnant; in CCHS 2005–2010, NA: age < 18 or pregnant

Table 1 presents characteristics of participants with arthritis from the 2001–2010 CCHS pooled together across all respondents (left-most column) and separately for those consulting each of the five types of Health Care Practitioner (HCP) under study. Among Canadians with arthritis in 2001–2010, 62% were female and 76% were age 50 years or older. Most were white (87%), non-immigrant (76%), had post-secondary education (44%) and were married (60%), while 35% were working in the past week and 34% had no job in the past week. The majority reported being non-smokers (78%), regular drinkers (51%), inactive (56%) and overweight (55%). Perceived general health was reported as poor for 9%, fair for 21%, good for 35%, very good for 26% and excellent for 9%. The distributions of these characteristics are similar when looking at the subsets who consulted the five types of HCP under study (columns 2–5).

Overall, in order of prevalence from highest to lowest, 92.0% (95%CI: 91.7–92.2%) of Canadians with arthritis consulted with a medical doctor, 14.5% (14.1–14.8%) with a physiotherapist, 14.2% (13.9–14.5%) with a nurse, 13.1% (12.8–13.4%) with a chiropractor and 3.0% (2.8–3.1%) with a psychologist in the previous 12 months (Table 1, row 2).

#### 2015–2018: regular healthcare provider (Table 2)

Table 2 presents characteristics of participants with arthritis from the 2015–2018 CCHS pooled together across all respondents (left-most column) and separately for those reporting receiving regular healthcare from each of the four types of HCP under study. Among Canadians with arthritis in 2015–2018, 59% were female and 83% were age 50 years or older. Most were white (82%), non-immigrant (77%), had post-secondary education (55%) and were married (55%), while 35% were working in the past week and 39% had no job in the past week. The majority reported being non-smokers (82%), regular drinkers (56%), active (46%) and overweight (60%). Perceived general health was reported as poor for 9%, fair for 18%, good for 35%, very good for 28% and excellent for 10%. The distributions of these characteristics are similar when looking at the subsets who received regular healthcare from the four types of HCP under study (columns 2–5).

**Table 2:** Characteristics of participants with arthritis and those who received regular health care from different health professionals: pooled analysis of CCHS 2015–2018

		Have received regular health care from a health professional				
Characteristics	Canadians with chronic arthritis	Medical doctor (incl. specialists)	Chiropractor	Physiotherapist	Nurse	Psychologist
	N (%)	n (%)	n (%)	n (%)	n (%)	n (%)
**Weighted sample**	5863028 (100.0%)	5335842 (100.0%)	563266 (100.0%)	551268 (100.0%)	441242 (100.0%)	226684 (100.0%)
**% Regular Health Care (95%CI)**		91.0 (90.5–91.5)	9.6 (9.1–10.1)	9.4 (8.9–9.9)	7.5 (7.2–7.9)	3.9 (3.5–4.2)
**Age group (years)**						
20–34	220737 (3.8%)	177532 (3.3%)	25943 (4.6%)	17745 (3.2%)	13163 (3.0%)	15815 (7.0%)
35–49	794676 (13.6%)	691606 (13.0%)	102353 (18.2%)	92962 (16.9%)	46866 (10.6%)	59486 (26.2%)
50–64	2145228 (36.6%)	1942068 (36.4%)	229745 (40.8%)	223170 (40.5%)	142801 (32.4%)	106958 (47.2%)
65–79	2056690 (35.1%)	1922312 (36.0%)	173421 (30.8%)	172010 (31.2%)	162822 (36.9%)	38684 (17.1%)
80+	645696 (11.0%)	602324 (11.3%)	31804 (5.6%)	45381 (8.2%)	75588 (17.1%)	5742 (2.5%)
**Sex**						
Male	2402957 (41.0%)	2163966 (40.6%)	235589 (41.8%)	198421 (36.0%)	156564 (35.5%)	84538 (37.3%)
Female	3460071 (59.0%)	3171877 (59.4%)	327677 (58.2%)	352847 (64.0%)	284678 (64.5%)	142146 (62.7%)
**Province of residence**						
Newfoundland and Labrador	123887 (2.1%)	114281 (2.1%)	7256 (1.3%)	8032 (1.5%)	10410 (2.4%)	3914 (1.7%)
Prince Edward Island	26216 (0.4%)	24075 (0.5%)	1049 (0.2%)	2863 (0.5%)	3897 (0.9%)	1020 (0.4%)
Nova Scotia	213473 (3.6%)	196682 (3.7%)	13377 (2.4%)	16936 (3.1%)	17468 (4.0%)	7937 (3.5%)
New Brunswick	151026 (2.6%)	141261 (2.6%)	4720 (0.8%)	9176 (1.7%)	14182 (3.2%)	4548 (2.0%)
Quebec	1253053 (21.4%)	1091826 (20.5%)	68821 (12.2%)	84395 (15.3%)	114346 (25.9%)	35900 (15.8%)
Ontario	2341325 (39.9%)	2173097 (40.7%)	246006 (43.7%)	252656 (45.8%)	190918 (43.3%)	101916 (45.0%)
Manitoba	202991 (3.5%)	186644 (3.5%)	22879 (4.1%)	23786 (4.3%)	13258 (3.0%)	8100 (3.6%)
Saskatchewan	174044 (3.0%)	156252 (2.9%)	24040 (4.3%)	16823 (3.1%)	18469 (4.2%)	7137 (3.1%)
Alberta	617725 (10.5%)	561122 (10.5%)	96546 (17.1%)	60154 (10.9%)	30437 (6.9%)	29057 (12.8%)
British Columbia	744388 (12.7%)	682416 (12.8%)	77902 (13.8%)	75360 (13.7%)	26001 (5.9%)	26551 (11.7%)
Yukon/Northwest Territories/Nunavut	14901 (0.3%)	8187 (0.2%)	669 (0.1%)	1087 (0.2%)	1855 (0.4%)	605 (0.3%)
**Cultural/racial background**						
White	4793160 (81.8%)	4417769 (82.8%)	491095 (87.2%)	444107 (80.6%)	382625 (86.7%)	190793 (84.2%)
Non-white	883621 (15.1%)	810065 (15.2%)	61406 (10.9%)	95559 (17.3%)	50985 (11.6%)	31517 (13.9%)
.	186247 (3.2%)	108008 (2.0%)	10764 (1.9%)	11603 (2.1%)	7632 (1.7%)	4374 (1.9%)
**Immigrant status**						
Non-immigrant	4501694 (76.8%)	4126239 (77.3%)	466697 (82.9%)	407342 (73.9%)	360207 (81.6%)	196336 (86.6%)
Immigrant(0–9 years)	91304 (1.6%)	80907 (1.5%)	9203 (1.6%)	6287 (1.1%)	8000 (1.8%)	1440 (0.6%)
Immigrant(10+ years)	1062175 (18.1%)	999180 (18.7%)	78134 (13.9%)	123503 (22.4%)	64295 (14.6%)	26414 (11.7%)
.	207854 (3.5%)	129515 (2.4%)	9231 (1.6%)	14137 (2.6%)	8740 (2.0%)	2494 (1.1%)
**Highest level of education - respondents**						
< secondary	1182717 (20.2%)	1063779 (19.9%)	70502 (12.5%)	61384 (11.1%)	106014 (24.0%)	27530 (12.1%)
Secondary grad	1357879 (23.2%)	1241065 (23.3%)	126908 (22.5%)	111408 (20.2%)	99290 (22.5%)	54916 (24.2%)
some post-sec edu	3194915 (54.5%)	2920029 (54.7%)	357358 (63.4%)	369281 (67.0%)	226939 (51.4%)	140160 (61.8%)
.	127517 (2.2%)	110970 (2.1%)	8498 (1.5%)	9195 (1.7%)	8999 (2.0%)	4079 (1.8%)
**Distribution of total household income**						
1st quintile	1327808 (22.6%)	1164287 (21.8%)	71476 (12.7%)	76037 (13.8%)	119888 (27.2%)	65558 (28.9%)
2nd quintile	1317710 (22.5%)	1187732 (22.3%)	100010 (17.8%)	97093 (17.6%)	103658 (23.5%)	45004 (19.9%)
3rd quintile	1162357 (19.8%)	1076970 (20.2%)	116195 (20.6%)	112021 (20.3%)	82043 (18.6%)	38183 (16.8%)
4th quintile	1002243 (17.1%)	928600 (17.4%)	127062 (22.6%)	122622 (22.2%)	64880 (14.7%)	39430 (17.4%)
5th quintile	1037047 (17.7%)	969250 (18.2%)	147768 (26.2%)	142409 (25.8%)	68917 (15.6%)	37904 (16.7%)
NA(residents of territories)*	14823 (0.3%)	8132 (0.2%)	669 (0.1%)	1087 (0.2%)	1844 (0.4%)	605 (0.3%)
.	1041 (0.0%)	872 (0.0%)	85 (0.0%)	0	12 (0.0%)	0
**Working status last week**						
Working	2041861 (34.8%)	1837721 (34.4%)	263530 (46.8%)	217284 (39.4%)	113285 (25.7%)	74831 (33.0%)
Absent	237414 (4.0%)	216422 (4.1%)	34236 (6.1%)	37870 (6.9%)	16003 (3.6%)	21292 (9.4%)
No job	2309371 (39.4%)	2138097 (40.1%)	187836 (33.3%)	205616 (37.3%)	189413 (42.9%)	117122 (51.7%)
NA(age < 15 or > 75)	1166662 (19.9%)	1092760 (20.5%)	71726 (12.7%)	81725 (14.8%)	119509 (27.1%)	12271 (5.4%)
.	107720 (1.8%)	50842 (1.0%)	5938 (1.1%)	8773 (1.6%)	3032 (0.7%)	1167 (0.5%)
**Marital status**						
Married	3211712 (54.8%)	2993924 (56.1%)	347338 (61.7%)	325620 (59.1%)	229821 (52.1%)	95124 (42.0%)
Common-law	543919 (9.3%)	470473 (8.8%)	56804 (10.1%)	52825 (9.6%)	34736 (7.9%)	26566 (11.7%)
Widowed/Divorced/Separated	1433173 (24.4%)	1306058 (24.5%)	105573 (18.7%)	119637 (21.7%)	127706 (28.9%)	60066 (26.5%)
Single	662294 (11.3%)	554638 (10.4%)	52461 (9.3%)	51707 (9.4%)	48542 (11.0%)	44711 (19.7%)
.	11930 (0.2%)	10749 (0.2%)	1089 (0.2%)	1480 (0.3%)	437 (0.1%)	218 (0.1%)
**Type of smoker**						
Daily	844093 (14.4%)	721287 (13.5%)	51520 (9.1%)	45719 (8.3%)	56370 (12.8%)	56760 (25.0%)
Occasional	187672 (3.2%)	165466 (3.1%)	15905 (2.8%)	14607 (2.6%)	12968 (2.9%)	14469 (6.4%)
Not at all	4829704 (82.4%)	4447649 (83.4%)	495797 (88.0%)	490886 (89.0%)	371581 (84.2%)	155340 (68.5%)
.	1560 (0.0%)	1440 (0.0%)	44 (0.0%)	56 (0.0%)	323 (0.1%)	115 (0.1%)
**Type of drinker**						
Regular	3285227 (56.0%)	2988042 (56.0%)	381781 (67.8%)	340784 (61.8%)	228233 (51.7%)	112659 (49.7%)
Occasional	1031643 (17.6%)	937532 (17.6%)	82690 (14.7%)	97603 (17.7%)	86914 (19.7%)	43274 (19.1%)
Did not drink	1511137 (25.8%)	1378967 (25.8%)	97069 (17.2%)	108588 (19.7%)	122737 (27.8%)	70172 (31.0%)
.	35020 (0.6%)	31301 (0.6%)	1725 (0.3%)	4294 (0.8%)	3357 (0.8%)	579 (0.3%)
**Physical activity**						
Active	2690520 (45.9%)	2439641 (45.7%)	308784 (54.8%)	292186 (53.0%)	183326 (41.5%)	113912 (50.3%)
Moderate active	1298389 (22.1%)	1190389 (22.3%)	121567 (21.6%)	108206 (19.6%)	93072 (21.1%)	51335 (22.6%)
Inactive	1727016 (29.5%)	1583740 (29.7%)	123335 (21.9%)	140436 (25.5%)	153103 (34.7%)	58181 (25.7%)
.	147103 (2.5%)	122072 (2.3%)	9580 (1.7%)	10440 (1.9%)	11740 (2.7%)	3257 (1.4%)
**BMI classification - International standard**						
Underweight	78270 (1.3%)	69417 (1.3%)	4331 (0.8%)	5560 (1.0%)	7935 (1.8%)	3488 (1.5%)
Normal weight	1740895 (29.7%)	1560612 (29.2%)	158258 (28.1%)	171069 (31.0%)	111543 (25.3%)	67400 (29.7%)
Overweight (incl. obese)	3533536 (60.3%)	3235539 (60.6%)	369525 (65.6%)	334430 (60.7%)	267838 (60.7%)	140627 (62.0%)
NA(age < 18 or pregnant)	2463 (0.0%)	1173 (0.0%)	177 (0.0%)	369 (0.1%)	0	0
.	507864 (8.7%)	469101 (8.8%)	30975 (5.5%)	39840 (7.2%)	53926 (12.2%)	15169 (6.7%)
**Perceived general health**						
Poor	513958 (8.8%)	469891 (8.8%)	33580 (6.0%)	53838 (9.8%)	65642 (14.9%)	48178 (21.3%)
Fair	1062300 (18.1%)	968973 (18.2%)	86752 (15.4%)	94238 (17.1%)	96104 (21.8%)	63259 (27.9%)
Good	2022344 (34.5%)	1841252 (34.5%)	196288 (34.8%)	187216 (34.0%)	154517 (35.0%)	70289 (31.0%)
Very good	1652009 (28.2%)	1501508 (28.1%)	183218 (32.5%)	157993 (28.7%)	94967 (21.5%)	33613 (14.8%)
Excellent	596328 (10.2%)	539863 (10.1%)	60272 (10.7%)	54490 (9.9%)	28002 (6.3%)	9857 (4.3%)
.	16088 (0.3%)	14355 (0.3%)	3155 (0.6%)	3493 (0.6%)	2010 (0.5%)	1488 (0.7%)
**CCHS survey cycle**						
CCHS 2015	3003318 (51.2%)	2717458 (50.9%)	283579 (50.3%)	272755 (49.5%)	230339 (52.2%)	106659 (47.1%)
CCHS 2016	2859710 (48.8%)	2618384 (49.1%)	279687 (49.7%)	278514 (50.5%)	210903 (47.8%)	120026 (52.9%)

1. Two cycles from Canadian Community Health Survey 2015–2018 were pooled to obtain a single sample representing an average population over these four years; Original sampling weights within each cycle were rescaled by dividing 2 (the number of survey cycles) to obtain a new weight in the newly pooled average population. 2. Sampling weights were used to produce Weighted number and percentage. 3. Data source: Public Use Microdata File of Canadian Community Health Survey produced by Statistics Canada and accessed via Ontario Data Documentation, Extraction Service and Infrastructure Initiative. 4. The results or view expressed are those of the authors and are not those of Statistics Canada.*: NA = not applicable according to population exclusions

Overall, in order of prevalence from highest to lowest, 91.0% (95%CI: 90.5–91.5%) of Canadians with arthritis received regular health care from a medical doctor, 9.6% (9.1–10.1%) from a chiropractor, 9.4% (8.9–9.9%) from a physiotherapist, 7.5% (7.2–7.9%) from a nurse and 3.9% (3.5–4.2%) from a psychologist in the previous 12 months (Table 2, row 2).

### Factors associated with health care utilization

Crude and adjusted prevalence ratios (PR) with 95% CIs from modified Poisson regression for consultation with each of the five HCPs across a range of factors are presented in Tables 3 and 4. Interpretations and results presented here will focus on the adjusted PRs.

**Table 3:** Regression analysis of the association between personal characteristics and self-reported consultations with health professionals among Canadians with chronic arthritis: pooled analysis of CCHS 2001–2010

Characteristics	Medical doctor (including specialists)		Physiotherapist		Nurse		Chiropractor		Psychologist	
	cPR (95%CI)^†^	aPR (95%CI)^††^	cPR (95%CI)	aPR (95%CI)	cPR (95%CI)	aPR (95%CI)	cPR(95%CI)	aPR(95%CI)	cPR(95%CI)	aPR(95%CI)
**Age group (years)**										
20–34	1.00	1.00	1.00	1.00	1.00	1.00	1.00	1.00	1.00	1.00
35–49	1.03(1.01–1.04)	1.00(0.98–1.02)	1.13(1.01–1.26)	1.04(0.92–1.17)	0.74(0.67–0.82)	0.70(0.63–0.78)	1.01(0.91–1.12)	0.98(0.88–1.09)	0.91(0.74–1.11)	0.95(0.77–1.18)
50–64	1.04(1.03–1.06)	1.01(0.99–1.03)	0.99(0.90–1.10)	0.90(0.81–1.01)	0.64(0.58–0.70)	0.55(0.50–0.61)	0.86(0.78–0.95)	0.86(0.78–0.96)	0.53(0.44–0.64)	0.54(0.43–0.67)
65–79	1.07(1.05–1.08)	1.02(1.00–1.04)	0.78(0.70–0.86)	0.82(0.72–0.93)	0.63(0.58–0.69)	0.49(0.44–0.54)	0.61(0.56–0.68)	0.76(0.67–0.85)	0.16(0.13–0.20)	0.15(0.11–0.20)
80+	1.07(1.06–1.09)	1.02(1.00–1.04)	0.68(0.60–0.76)	0.81(0.68–0.95)	0.99(0.90–1.09)	0.65(0.57–0.75)	0.40(0.36–0.45)	0.59(0.50–0.69)	0.13(0.10–0.17)	0.15(0.09–0.24)
**Sex**										
Male	1.00	1.00	1.00	1.00	1.00	1.00	1.00	1.00	1.00	1.00
Female	1.04(1.03–1.04)	1.04(1.03–1.05)	1.08(1.03–1.14)	1.21(1.15–1.28)	1.13(1.08–1.18)	1.11(1.06–1.17)	0.98(0.94–1.03)	1.09(1.04–1.15)	1.12(0.99–1.26)	1.18(1.04–1.35)
**Province of residence**										
Newfoundland and Labrador	1.01(1.00–1.02)	1.02(1.01–1.03)	0.67(0.59–0.76)	0.73(0.64–0.83)	1.03(0.93–1.14)	0.98(0.87–1.09)	0.43(0.36–0.51)	0.41(0.34–0.50)	0.76(0.54–1.09)	0.84(0.58–1.20)
Prince Edward Island	1.01(1.00–1.02)	1.02(1.00–1.03)	0.92(0.79–1.08)	1.04(0.88–1.21)	1.07(0.93–1.22)	0.96(0.84–1.10)	0.38(0.30–0.49)	0.35(0.27–0.46)	0.72(0.43–1.20)	0.80(0.48–1.34)
Nova Scotia	1.01(1.00–1.02)	1.01(1.00–1.03)	0.95(0.86–1.04)	0.98(0.88–1.08)	0.87(0.79–0.97)	0.78(0.70–0.87)	0.41(0.36–0.48)	0.40(0.34–0.46)	1.31(1.03–1.67)	1.22(0.94–1.59)
New Brunswick	1.00(0.99–1.01)	1.00(0.99–1.01)	0.81(0.73–0.90)	0.87(0.78–0.97)	1.17(1.07–1.28)	0.99(0.90–1.09)	0.53(0.46–0.62)	0.53(0.46–0.62)	1.15(0.91–1.44)	0.98(0.77–1.24)
Quebec	0.98(0.97–0.98)	0.98(0.97–0.99)	0.78(0.72–0.84)	0.86(0.79–0.93)	1.49(1.41–1.58)	1.43(1.35–1.52)	0.67(0.62–0.72)	0.70(0.64–0.75)	1.71(1.47–2.00)	1.82(1.56–2.13)
Ontario	1.00	1.00	1.00	1.00	1.00	1.00	1.00	1.00	1.00	1.00
Manitoba	0.99(0.98–1.00)	0.99(0.98–1.00)	1.11(1.02–1.22)	1.18(1.07–1.30)	0.91(0.82–1.01)	0.87(0.78–0.98)	1.49(1.37–1.63)	1.43(1.31–1.57)	0.97(0.74–1.28)	1.05(0.78–1.40)
Saskatchewan	0.99(0.99–1.00)	1.00(0.99–1.01)	0.92(0.83–1.01)	0.97(0.88–1.07)	1.06(0.97–1.15)	0.98(0.90–1.07)	1.40(1.29–1.51)	1.31(1.21–1.42)	0.81(0.63–1.03)	0.85(0.66–1.10)
Alberta	0.99(0.98–1.00)	0.99(0.99–1.00)	1.11(1.03–1.20)	1.10(1.01–1.19)	1.11(1.02–1.20)	1.10(1.01–1.19)	1.58(1.47–1.69)	1.46(1.36–1.56)	1.55(1.29–1.85)	1.59(1.32–1.91)
British Columbia	1.00(1.00–1.01)	1.00(1.00–1.01)	1.24(1.16–1.32)	1.22(1.14–1.30)	0.86(0.80–0.92)	0.85(0.79–0.92)	1.19(1.11–1.27)	1.14(1.07–1.22)	1.12(0.92–1.37)	1.19(0.97–1.47)
Yukon/Northwest Territories/Nunavut	0.94(0.92–0.96)	0.97(0.94–1.00)	1.14(0.99–1.32)	1.15(0.99–1.35)	2.71(2.47–2.97)	2.82(2.52–3.16)	0.76(0.63–0.92)	0.69(0.57–0.84)	1.50(1.03–2.18)	1.36(0.90–2.04)
**Cultural/racial background**										
White	1.00	1.00	1.00	1.00	1.00	1.00	1.00	1.00	1.00	1.00
Non-white	1.00(0.99–1.01)	0.99(0.98–1.01)	1.15(1.04–1.26)	1.12(1.02–1.23)	0.73(0.66–0.81)	0.84(0.75–0.93)	0.83(0.74–0.93)	0.88(0.78–0.98)	1.15(0.91–1.45)	0.99(0.80–1.23)
**Immigrant status**										
Non-immigrant	1.00	1.00	1.00	1.00	1.00	1.00	1.00	1.00	1.00	1.00
Immigrant(0–9 years)	1.02(1.00–1.05)	1.02(0.99–1.05)	1.02(0.76–1.37)	0.89(0.64–1.24)	0.41(0.28–0.59)	0.35(0.23–0.54)	0.82(0.58–1.17)	0.88(0.62–1.26)	0.92(0.32–2.64)	0.69(0.22–2.20)
Immigrant(10+ years)	1.02(1.01–1.03)	1.01(1.00–1.01)	1.04(0.97–1.11)	0.99(0.93–1.05)	0.68(0.64–0.73)	0.72(0.67–0.78)	0.75(0.69–0.80)	0.77(0.71–0.83)	0.66(0.55–0.79)	0.86(0.72–1.04)
**Highest level of education - respondents**										
< secondary	0.99(0.99–1.00)	0.97(0.97–0.98)	0.53(0.51–0.57)	0.64(0.60–0.68)	0.94(0.90–0.99)	0.77(0.73–0.82)	0.62(0.59–0.65)	0.86(0.81–0.91)	0.47(0.41–0.53)	0.55(0.47–0.63)
Secondary grad	0.99(0.99–1.00)	0.99(0.98–1.00)	0.76(0.72–0.81)	0.80(0.75–0.86)	0.82(0.77–0.87)	0.80(0.75–0.85)	0.84(0.79–0.90)	0.90(0.84–0.97)	0.67(0.56–0.78)	0.69(0.58–0.81)
some post-sec edu	1.00(0.99–1.01)	1.00(0.99–1.01)	0.80(0.74–0.88)	0.83(0.75–0.91)	1.07(0.98–1.17)	1.00(0.91–1.09)	0.97(0.89–1.06)	1.02(0.93–1.11)	0.97(0.81–1.17)	0.84(0.70–1.02)
Post-sec grad/university degree	1.00	1.00	1.00	1.00	1.00	1.00	1.00	1.00	1.00	1.00
**Total household income**										
1st quintile	0.99(0.98–1.00)	0.96(0.95–0.97)	0.57(0.52–0.62)	0.60(0.55–0.67)	1.39(1.29–1.50)	0.97(0.89–1.07)	0.47(0.43–0.52)	0.72(0.65–0.80)	1.15(0.95–1.39)	0.88(0.71–1.10)
2nd quintile	0.99(0.98–1.00)	0.97(0.96–0.98)	0.63(0.58–0.69)	0.69(0.63–0.76)	1.14(1.06–1.24)	0.93(0.85–1.01)	0.65(0.60–0.71)	0.91(0.83–0.99)	0.88(0.73–1.08)	0.86(0.70–1.06)
3rd quintile	1.00(0.99–1.01)	0.98(0.97–0.99)	0.67(0.62–0.72)	0.75(0.69–0.81)	1.20(1.10–1.30)	1.05(0.96–1.15)	0.75(0.70–0.81)	1.00(0.92–1.08)	0.81(0.67–0.98)	0.82(0.68–1.00)
4th quintile	1.00(0.99–1.00)	0.99(0.98–1.00)	0.83(0.77–0.89)	0.89(0.83–0.96)	1.03(0.96–1.12)	0.98(0.90–1.06)	0.90(0.84–0.96)	1.04(0.97–1.12)	0.91(0.75–1.10)	0.89(0.73–1.09)
5th quintile	1.00	1.00	1.00	1.00	1.00	1.00	1.00	1.00	1.00	1.00
NA/NS*	0.99(0.98–1.00)	0.97(0.96–0.98)	0.64(0.60–0.69)	0.72(0.66–0.79)	1.00(0.92–1.08)	0.84(0.76–0.92)	0.64(0.60–0.69)	0.92(0.85–1.01)	0.61(0.50–0.75)	0.68(0.54–0.86)
**Working status last week**										
Working	1.00	1.00	1.00	1.00	1.00	1.00	1.00	1.00	1.00	1.00
Absent	1.03(1.01–1.05)	1.02(1.01–1.04)	1.50(1.36–1.65)	1.44(1.31–1.59)	1.57(1.40–1.75)	1.40(1.25–1.58)	0.99(0.89–1.11)	1.02(0.91–1.14)	1.77(1.44–2.17)	1.54(1.25–1.89)
No job	1.03(1.03–1.04)	1.02(1.02–1.03)	0.82(0.77–0.86)	1.00(0.94–1.07)	1.10(1.03–1.16)	1.12(1.04–1.19)	0.64(0.61–0.67)	0.81(0.75–0.86)	0.88(0.76–1.01)	1.34(1.13–1.58)
Unable/permanent	1.07(1.06–1.08)	1.05(1.04–1.06)	1.08(0.98–1.18)	1.24(1.12–1.38)	1.88(1.74–2.04)	1.36(1.24–1.49)	0.54(0.48–0.61)	0.77(0.67–0.88)	1.90(1.61–2.23)	1.62(1.32–1.99)
NA(age > 75)	1.05(1.05–1.06)	1.03(1.02–1.04)	0.69(0.65–0.74)	0.96(0.86–1.06)	1.44(1.36–1.52)	1.23(1.11–1.36)	0.44(0.41–0.47)	0.69(0.62–0.77)	0.24(0.20–0.30)	0.91(0.64–1.29)
**Marital status**										
Married	1.00	1.00	1.00	1.00	1.00	1.00	1.00	1.00	1.00	1.00
Common-law	0.96(0.95–0.97)	0.98(0.97–1.00)	1.01(0.92–1.12)	1.01(0.91–1.12)	1.22(1.11–1.34)	1.00(0.90–1.10)	1.04(0.95–1.14)	1.00(0.90–1.11)	2.38(1.87–3.03)	1.41(1.11–1.79)
Widowed/Divorced/Separated	1.00(1.00–1.01)	0.99(0.99–1.00)	0.84(0.80–0.88)	0.99(0.94–1.05)	1.32(1.26–1.38)	1.15(1.09–1.21)	0.77(0.73–0.81)	0.99(0.94–1.06)	1.37(1.21–1.55)	1.67(1.44–1.94)
Single	0.96(0.95–0.97)	0.98(0.97–0.99)	0.93(0.86–1.00)	0.96(0.89–1.04)	1.35(1.26–1.45)	1.07(1.00–1.16)	0.86(0.79–0.92)	0.86(0.79–0.93)	2.53(2.19–2.92)	1.53(1.30–1.81)
**Type of smoker**										
Daily	0.95(0.95–0.96)	0.96(0.95–0.97)	0.81(0.76–0.87)	0.79(0.73–0.85)	0.97(0.92–1.02)	0.83(0.78–0.88)	0.84(0.79–0.90)	0.76(0.71–0.82)	1.71(1.52–1.92)	0.94(0.83–1.07)
Occasional	0.97(0.95–0.99)	0.98(0.96–1.00)	0.93(0.80–1.09)	0.83(0.71–0.98)	1.04(0.91–1.17)	0.94(0.83–1.07)	1.05(0.92–1.19)	0.87(0.76–1.00)	2.24(1.61–3.11)	1.34(0.97–1.87)
Not at all	1.00	1.00	1.00	1.00	1.00	1.00	1.00	1.00	1.00	1.00
**Type of drinker**										
Regular	0.99(0.98–0.99)	1.01(1.00–1.02)	1.25(1.18–1.33)	1.16(1.09–1.23)	0.81(0.77–0.85)	0.93(0.88–0.98)	1.49(1.40–1.58)	1.14(1.07–1.22)	1.10(0.95–1.26)	1.01(0.88–1.16)
Occasional	1.00(1.00–1.01)	1.01(1.00–1.02)	1.06(0.99–1.13)	1.02(0.95–1.09)	1.01(0.95–1.07)	1.04(0.98–1.10)	1.27(1.18–1.36)	1.08(1.00–1.16)	1.03(0.88–1.20)	0.92(0.78–1.08)
Did not drink	1.00	1.00	1.00	1.00	1.00	1.00	1.00	1.00	1.00	1.00
**Physical activity**										
Active	1.00	1.00	1.00	1.00	1.00	1.00	1.00	1.00	1.00	1.00
Moderate active	1.02(1.01–1.03)	1.01(1.00–1.02)	0.94(0.88–1.01)	0.93(0.87–1.00)	1.06(0.99–1.14)	1.02(0.95–1.09)	0.95(0.89–1.01)	0.99(0.93–1.06)	1.02(0.86–1.21)	1.07(0.89–1.28)
Inactive	1.03(1.02–1.03)	1.01(1.00–1.02)	0.82(0.78–0.87)	0.87(0.82–0.93)	1.17(1.10–1.24)	0.98(0.92–1.05)	0.78(0.74–0.83)	0.93(0.87–0.98)	1.05(0.90–1.21)	1.09(0.94–1.28)
**BMI classification - International standard**										
Underweight	1.01(0.99–1.03)	1.00(0.98–1.02)	0.92(0.79–1.07)	0.92(0.79–1.06)	1.45(1.28–1.64)	1.17(1.03–1.33)	0.85(0.72–1.00)	0.97(0.82–1.15)	1.37(1.05–1.78)	1.01(0.76–1.33)
Normal weight	1.00	1.00	1.00	1.00	1.00	1.00	1.00	1.00	1.00	1.00
Overweight (incl. obese)	1.01(1.01–1.02)	1.01(1.00–1.01)	1.03(0.98–1.09)	1.02(0.97–1.08)	1.06(1.01–1.11)	1.04(0.99–1.10)	1.06(1.01–1.12)	1.04(0.99–1.09)	0.88(0.78–1.00)	0.90(0.79–1.02)
NA (pregnant)**	1.04(1.03–1.05)	1.01(1.00–1.02)	0.81(0.74–0.88)	1.08(0.97–1.20)	1.10(1.02–1.19)	1.25(1.14–1.37)	0.76(0.69–0.83)	1.03(0.92–1.15)	0.30(0.23–0.40)	1.16(0.83–1.62)
**Perceived general health**										
Poor	1.12(1.10–1.13)	1.12(1.10–1.13)	1.46(1.30–1.63)	2.01(1.78–2.27)	2.89(2.59–3.23)	2.93(2.60–3.30)	0.68(0.60–0.77)	1.08(0.94–1.23)	3.03(2.38–3.86)	3.46(2.62–4.55)
Fair	1.09(1.08–1.10)	1.09(1.08–1.11)	1.25(1.13–1.39)	1.70(1.52–1.89)	2.05(1.85–2.28)	2.11(1.89–2.36)	0.80(0.73–0.88)	1.12(1.02–1.24)	1.94(1.53–2.47)	2.67(2.06–3.45)
Good	1.06(1.05–1.08)	1.06(1.05–1.08)	1.17(1.06–1.29)	1.39(1.26–1.53)	1.47(1.32–1.63)	1.52(1.36–1.70)	0.94(0.86–1.02)	1.11(1.01–1.21)	1.38(1.09–1.75)	1.73(1.35–2.21)
Very good	1.04(1.03–1.05)	1.04(1.03–1.05)	1.16(1.05–1.28)	1.21(1.09–1.33)	1.19(1.07–1.34)	1.24(1.10–1.39)	1.02(0.94–1.12)	1.06(0.97–1.16)	1.15(0.90–1.46)	1.27(0.99–1.64)
Excellent	1.00	1.00	1.00	1.00	1.00	1.00	1.00	1.00	1.00	1.00
**CCHS survey cycle**										
every two-year increase	1.00(1.00–1.00)	1.00(1.00–1.00)	1.04(1.03–1.06)	1.06(1.04–1.08)	1.06(1.04–1.07)	1.09(1.07–1.11)	1.00(0.98–1.01)	1.01(1.00–1.03)	1.04(0.99–1.08)	1.06(1.01–1.12)

1. Data source: Public Use Microdata File of Canadian Community Health Survey produced by Statistics Canada and accessed via Ontario Data Documentation, Extraction Service and Infrastructure Initiative. 2. Five cycles from Canadian Community Health Survey 2001–2010 were pooled to obtain a single sample representing an average population over these ten years; Original sampling weights within each cycle were rescaled by dividing 5 (the number of survey cycles) to obtain a new weight in the newly pooled average population. Missing data on any of the independent and dependent variables were dropped from analysis in the final model. All statistics are based on the rescaled sampling weights. The results or view expressed are those of the authors and are not those of Statistics Canada. ^†^: cPR (95%CI): crude prevalence ratio and 95% confidence interval were obtained from bivariate Poisson regression (with robust error variance estimate) between the consultation with a specific health care provider (yes or no) and each of the personal characteristics. ^††^: aPR (95%CI): prevalence ratio and 95% confidence interval were obtained from multivariable Poisson regression (with robust error variance estimate). All of the 14 personal characteristics listed in the table were entered in the multivariable regression model. *: NA = not applicable according to population exclusions. NS = not stated or responses without enough information for classification. For Total Household Income, Not Stated was merged with Not Applicable and treated as one category together. **: In CCHS 2001, NA: age > 64 or pregnant

**Table 4:** Regression analysis of the association between personal characteristics and regular health care utilization among Canadians with chronic arthritis: CCHS 2015–2018

Characteristics	Medical doctor (including specialist)		Chiropractor		Physiotherapist		Nurse		Psychologist	
	**cPR (95%CI)**^**†**^	**aPR (95%CI)**^**††**^	cPR (95%CI)	aPR (95%CI)	cPR (95%CI)	aPR (95%CI)	cPR(95%CI)	aPR(95%CI)	cPR(95%CI)	aPR(95%CI)
**Age group (years)**										
20–34	1.00	1.00	1.00	1.00	1.00	1.00	1.00	1.00	1.00	1.00
35–49	1.09(1.05–1.14)	1.08(1.03–1.13)	1.11(0.86–1.43)	1.05(0.80–1.39)	1.47(1.06–2.05)	1.31(0.91–1.87)	1.00(0.70–1.42)	1.03(0.71–1.49)	1.05(0.76–1.47)	1.24(0.87–1.79)
50–64	1.14(1.09–1.19)	1.11(1.06–1.17)	0.92(0.74–1.15)	0.89(0.69–1.14)	1.31(0.96–1.78)	1.16(0.82–1.63)	1.13(0.81–1.57)	1.11(0.79–1.57)	0.70(0.52–0.95)	0.77(0.55–1.06)
65–79	1.17(1.13–1.22)	1.14(1.08–1.19)	0.73(0.58–0.91)	0.84(0.65–1.09)	1.05(0.78–1.42)	1.06(0.75–1.51)	1.34(0.97–1.86)	1.21(0.86–1.72)	0.27(0.19–0.37)	0.33(0.22–0.50)
80+	1.18(1.13–1.23)	1.14(1.09–1.20)	0.43(0.33–0.55)	0.63(0.46–0.88)	0.89(0.64–1.23)	1.08(0.71–1.63)	1.99(1.44–2.76)	1.70(1.17–2.46)	0.13(0.08–0.21)	0.23(0.10–0.51)
**Sex**										
Male	1.00	1.00	1.00	1.00	1.00	1.00	1.00	1.00	1.00	1.00
Female	1.02(1.01–1.03)	1.03(1.01–1.04)	0.97(0.88–1.06)	1.16(1.06–1.27)	1.24(1.12–1.37)	1.30(1.17–1.44)	1.27(1.16–1.39)	1.24(1.12–1.38)	1.17(0.96–1.43)	1.23(1.00–1.51)
Province of residence*										
Newfoundland and Labrador	0.97(0.96–0.99)	0.97(0.95–0.99)	0.55(0.43–0.69)	0.55(0.43–0.69)	0.59(0.45–0.77)	0.60(0.46–0.79)	1.01(0.81–1.26)	0.92(0.72–1.17)	0.71(0.49–1.03)	0.66(0.44–0.99)
Prince Edward Island	0.97(0.95–0.99)	0.96(0.94–0.99)	0.37(0.25–0.55)	0.37(0.25–0.56)	0.99(0.75–1.32)	1.09(0.81–1.47)	1.79(1.50–2.15)	1.51(1.23–1.86)	0.88(0.59–1.32)	0.85(0.55–1.32)
Nova Scotia	0.97(0.96–0.99)	0.97(0.95–0.99)	0.58(0.46–0.74)	0.53(0.41–0.69)	0.72(0.58–0.89)	0.74(0.60–0.93)	0.98(0.84–1.14)	0.89(0.76–1.05)	0.84(0.62–1.13)	0.70(0.50–0.98)
New Brunswick	0.99(0.97–1.01)	0.98(0.96–1.00)	0.29(0.22–0.38)	0.26(0.19–0.35)	0.55(0.44–0.69)	0.60(0.47–0.76)	1.13(0.93–1.37)	1.01(0.81–1.25)	0.68(0.48–0.97)	0.55(0.38–0.79)
Quebec	0.92(0.91–0.93)	0.93(0.92–0.94)	0.51(0.45–0.58)	0.49(0.43–0.56)	0.61(0.53–0.70)	0.70(0.61–0.81)	1.10(0.99–1.22)	1.00(0.89–1.13)	0.65(0.52–0.80)	0.70(0.54–0.89)
Ontario	1.00	1.00	1.00	1.00	1.00	1.00	1.00	1.00	1.00	1.00
Manitoba	0.97(0.95–0.99)	0.98(0.96–1.00)	1.05(0.90–1.23)	1.03(0.87–1.22)	1.06(0.87–1.30)	1.15(0.92–1.43)	0.78(0.65–0.95)	0.79(0.65–0.98)	0.90(0.57–1.40)	0.88(0.56–1.36)
Saskatchewan	0.95(0.93–0.96)	0.93(0.91–0.96)	1.28(1.09–1.52)	1.12(0.94–1.34)	0.87(0.71–1.07)	0.91(0.73–1.13)	1.27(1.09–1.49)	1.12(0.95–1.32)	0.92(0.62–1.37)	0.84(0.56–1.28)
Alberta	0.97(0.95–0.98)	0.97(0.95–0.98)	1.47(1.30–1.66)	1.32(1.15–1.51)	0.89(0.77–1.03)	0.90(0.77–1.05)	0.60(0.50–0.71)	0.63(0.52–0.76)	1.07(0.81–1.41)	0.98(0.73–1.32)
British Columbia	0.97(0.96–0.98)	0.97(0.96–0.98)	0.98(0.85–1.13)	0.93(0.81–1.08)	0.92(0.80–1.07)	0.92(0.79–1.08)	0.42(0.35–0.51)	0.41(0.32–0.51)	0.81(0.60–1.08)	0.80(0.59–1.08)
**Cultural/racial background**										
White	1.00	1.00	1.00	1.00	1.00	1.00	1.00	1.00	1.00	1.00
Non-white	1.00(0.98–1.01)	1.02(1.00–1.03)	0.68(0.57–0.80)	0.79(0.66–0.94)	1.17(0.98–1.40)	1.20(1.00–1.43)	0.72(0.61–0.86)	0.85(0.69–1.04)	0.90(0.68–1.18)	0.83(0.62–1.12)
**Immigrant status**										
Non-immigrant	1.00	1.00	1.00	1.00	1.00	1.00	1.00	1.00	1.00	1.00
Immigrant(0–9 years)	0.97(0.91–1.03)	0.96(0.91–1.02)	0.98(0.49–1.94)	1.21(0.61–2.40)	0.76(0.40–1.44)	0.70(0.32–1.53)	1.10(0.57–2.12)	1.35(0.62–2.93)	0.36(0.06–2.32)	0.39(0.06–2.63)
Immigrant(10+ years)	1.03(1.01–1.04)	0.99(0.97–1.00)	0.71(0.61–0.83)	0.73(0.62–0.87)	1.29(1.11–1.48)	1.13(0.98–1.30)	0.76(0.65–0.88)	0.75(0.63–0.90)	0.57(0.42–0.77)	0.65(0.47–0.89)
**Highest level of education - respondents**										
< secondary	0.99(0.98–1.00)	0.98(0.97–1.00)	0.54(0.47–0.61)	0.87(0.74–1.01)	0.45(0.38–0.54)	0.58(0.47–0.71)	1.27(1.15–1.40)	0.87(0.77–0.98)	0.53(0.41–0.69)	0.53(0.40–0.69)
Secondary grad	1.00(0.99–1.01)	1.00(0.99–1.01)	0.84(0.75–0.94)	0.92(0.82–1.03)	0.71(0.63–0.80)	0.80(0.71–0.91)	1.03(0.93–1.15)	0.97(0.86–1.09)	0.92(0.74–1.15)	0.85(0.67–1.08)
some post-sec education	1.00	1.00	1.00	1.00	1.00	1.00	1.00	1.00	1.00	1.00
**Distribution of total household income**										
1st quintile	0.96(0.94–0.97)	0.97(0.95–0.98)	0.39(0.33–0.45)	0.65(0.53–0.79)	0.43(0.36–0.51)	0.52(0.43–0.64)	1.39(1.20–1.60)	1.06(0.89–1.27)	1.38(1.11–1.72)	0.89(0.66–1.20)
2nd quintile	0.98(0.97–0.99)	0.98(0.96–0.99)	0.54(0.48–0.62)	0.78(0.67–0.90)	0.55(0.47–0.63)	0.62(0.53–0.73)	1.20(1.04–1.39)	0.92(0.79–1.08)	0.95(0.75–1.21)	0.92(0.70–1.20)
3rd quintile	0.99(0.98–1.01)	0.99(0.98–1.00)	0.70(0.62–0.80)	0.86(0.75–0.99)	0.70(0.61–0.81)	0.78(0.67–0.91)	1.07(0.91–1.24)	0.87(0.74–1.03)	0.90(0.69–1.18)	0.79(0.58–1.08)
4th quintile	0.99(0.98–1.00)	0.99(0.98–1.00)	0.89(0.78–1.02)	0.99(0.86–1.13)	0.89(0.77–1.03)	0.91(0.78–1.05)	0.97(0.82–1.15)	0.87(0.73–1.05)	1.08(0.79–1.48)	0.94(0.67–1.32)
5th quintile	1.00	1.00	1.00	1.00	1.00	1.00	1.00	1.00	1.00	1.00
**Working status last week**										
Working	1.00	1.00	1.00	1.00	1.00	1.00	1.00	1.00	1.00	1.00
Absent	1.01(0.99–1.04)	1.01(0.98–1.04)	1.12(0.94–1.34)	1.14(0.94–1.38)	1.50(1.26–1.79)	1.46(1.21–1.76)	1.22(0.92–1.62)	1.07(0.79–1.45)	2.45(1.87–3.20)	2.07(1.58–2.72)
No job	1.03(1.02–1.04)	1.01(1.00–1.02)	0.63(0.57–0.70)	0.80(0.71–0.90)	0.84(0.75–0.93)	1.02(0.90–1.15)	1.48(1.31–1.67)	1.18(1.03–1.36)	1.39(1.12–1.71)	1.51(1.18–1.94)
NA(age > 75)	1.06(1.05–1.07)	1.02(1.00–1.03)	0.48(0.43–0.55)	0.81(0.66–0.98)	0.67(0.59–0.76)	0.85(0.70–1.02)	1.88(1.67–2.12)	1.29(1.07–1.56)	0.29(0.21–0.40)	0.66(0.42–1.04)
**Marital status**										
Married	1.00	1.00	1.00	1.00	1.00	1.00	1.00	1.00	1.00	1.00
Common-law	0.92(0.90–0.94)	0.96(0.94–0.98)	0.96(0.80–1.15)	0.99(0.81–1.20)	0.95(0.79–1.15)	1.01(0.83–1.23)	0.89(0.75–1.05)	0.98(0.81–1.20)	1.64(1.20–2.25)	1.15(0.79–1.66)
Widowed/Divorced/Separated	0.98(0.97–0.99)	0.98(0.96–0.99)	0.68(0.62–0.76)	0.99(0.88–1.12)	0.83(0.73–0.93)	1.09(0.93–1.26)	1.25(1.14–1.37)	0.95(0.85–1.06)	1.42(1.15–1.75)	1.54(1.22–1.94)
Single	0.89(0.87–0.91)	0.93(0.91–0.95)	0.73(0.63–0.84)	0.82(0.70–0.95)	0.77(0.66–0.89)	0.93(0.79–1.09)	1.02(0.88–1.18)	1.00(0.83–1.20)	2.27(1.84–2.80)	1.33(1.04–1.70)
**Type of smoker**										
Daily	0.92(0.91–0.94)	0.95(0.93–0.97)	0.59(0.51–0.69)	0.59(0.50–0.69)	0.53(0.42–0.67)	0.49(0.40–0.59)	0.86(0.76–0.98)	0.87(0.75–1.00)	2.08(1.71–2.53)	1.16(0.94–1.44)
Occasional	0.96(0.93–0.98)	0.98(0.95–1.01)	0.82(0.66–1.03)	0.71(0.56–0.91)	0.76(0.59–0.99)	0.75(0.57–0.98)	0.90(0.68–1.19)	0.87(0.62–1.21)	2.39(1.68–3.42)	1.48(0.98–2.23)
Not at all	1.00	1.00	1.00	1.00	1.00	1.00	1.00	1.00	1.00	1.00
**Type of drinker**										
Regular	0.99(0.98–1.00)	0.99(0.98–1.01)	1.79(1.60–2.00)	1.39(1.24–1.57)	1.43(1.25–1.63)	1.27(1.10–1.47)	0.85(0.76–0.94)	1.09(0.97–1.23)	0.73(0.60–0.89)	0.67(0.52–0.85)
Occasional	0.99(0.98–1.00)	1.00(0.98–1.01)	1.24(1.08–1.43)	1.07(0.92–1.24)	1.31(1.08–1.59)	1.17(0.96–1.42)	1.03(0.91–1.17)	1.11(0.97–1.26)	0.90(0.71–1.14)	0.63(0.49–0.81)
Did not drink	1.00	1.00	1.00	1.00	1.00	1.00	1.00	1.00	1.00	1.00
**Physical activity**										
Active	1.00	1.00	1.00	1.00	1.00	1.00	1.00	1.00	1.00	1.00
Moderate active	1.02(1.00–1.03)	1.01(1.00–1.02)	0.82(0.73–0.92)	0.94(0.83–1.06)	0.77(0.68–0.87)	0.82(0.72–0.93)	1.06(0.94–1.18)	0.90(0.80–1.02)	0.94(0.76–1.16)	0.92(0.74–1.16)
Inactive	1.02(1.01–1.03)	1.00(0.99–1.01)	0.63(0.57–0.69)	0.91(0.81–1.02)	0.75(0.67–0.85)	0.87(0.76–1.00)	1.31(1.19–1.44)	0.89(0.80–1.00)	0.80(0.65–0.99)	0.90(0.70–1.14)
**BMI classification - International standard**										
Underweight	0.99(0.95–1.02)	1.00(0.96–1.04)	0.61(0.33–1.12)	0.71(0.38–1.32)	0.72(0.41–1.27)	0.83(0.47–1.44)	1.58(1.04–2.41)	1.22(0.79–1.87)	1.15(0.51–2.62)	0.78(0.33–1.84)
Normal weight	1.00	1.00	1.00	1.00	1.00	1.00	1.00	1.00	1.00	1.00
Overweight (incl. obese)	1.02(1.01–1.03)	1.02(1.01–1.03)	1.15(1.04–1.27)	1.08(0.97–1.19)	0.96(0.87–1.07)	0.93(0.84–1.04)	1.18(1.07–1.31)	1.17(1.06–1.30)	1.03(0.86–1.23)	0.91(0.76–1.08)
**Perceived general health**										
Poor	1.02(1.00–1.04)	1.04(1.02–1.07)	0.66(0.51–0.84)	1.09(0.82–1.46)	1.16(0.93–1.45)	1.88(1.46–2.42)	2.76(2.21–3.45)	2.50(1.96–3.19)	5.75(3.63–9.12)	4.89(2.97–8.04)
Fair	1.01(0.99–1.03)	1.02(1.00–1.05)	0.81(0.67–0.98)	1.25(1.01–1.54)	0.97(0.80–1.18)	1.50(1.21–1.85)	1.93(1.56–2.39)	1.85(1.48–2.32)	3.61(2.27–5.75)	3.80(2.34–6.18)
Good	1.01(0.99–1.03)	1.02(1.00–1.04)	0.96(0.81–1.14)	1.20(1.01–1.43)	1.02(0.85–1.22)	1.33(1.09–1.62)	1.64(1.34–2.00)	1.58(1.28–1.94)	2.11(1.31–3.41)	2.31(1.40–3.81)
Very good	1.01(0.99–1.02)	1.01(0.99–1.03)	1.10(0.93–1.30)	1.13(0.95–1.35)	1.05(0.86–1.27)	1.14(0.94–1.39)	1.23(0.99–1.51)	1.13(0.91–1.41)	1.23(0.77–1.96)	1.23(0.76–2.01)
Excellent	1.00	1.00	1.00	1.00	1.00	1.00	1.00	1.00	1.00	1.00

3. Data source: Public Use Microdata File of Canadian Community Health Survey produced by Statistics Canada and accessed via Ontario Data Documentation, Extraction Service and Infrastructure Initiative. 4. Two cycles from Canadian Community Health Survey 2015–2016 were pooled to obtain a single sample representing an average population over these four years; Original sampling weights within each cycle were rescaled by dividing 2 (the number of survey cycles) to obtain a new weight in the newly pooled average population. Missing data on any of the independent and dependent variables were dropped from analysis in the final model. All statistics are based on sampling weights and 500 bootstrap weights. The results or view expressed are those of the authors and are not those of Statistics Canada. ^†^: cPR (95%CI): crude prevalence ratio and 95% confidence interval were obtained from bivariate Poisson regression (with robust error variance estimate) between the consultation with a specific health care provider (yes or no) and each of the personal characteristics. ^††^: aPR (95%CI): prevalence ratio and 95% confidence interval were obtained from multivariable Poisson regression (with robust error variance estimate). All of the 14 personal characteristics listed in the table were entered in the multivariable regression model. * Participants from territories were excluded from final model because their income information was unavailable

#### CCHS 2001–2010: consultations with HCP (Table 3)

Across all HCPs, females were more likely to consult a provider than males with PRs ranging from 1.04 (95%CI: 1.03–1.04) for medical doctor to 1.21 (95%CI: 1.15–1.28) for physiotherapist.

**Medical Doctor** Overall, prevalence ratios across most factors were close to 1.0 indicating small variation across most factors in who consulted a medical doctor over the past 12 months. *Predisposing Factors* There was a mild gradient across age, with older ages more likely to consult a medical doctor than younger ages with PR 1.02 (95%CI: 1.00–1.04) for the oldest age group compared with the youngest. *Enabling Factors* For working status, compared to those working, other working statuses were more likely to consult with a medical doctor with the highest PR for those permanently unable to work (PR 1.05 (95%CI: 1.04–1.06)). *Need Factors* There was a stronger gradient across levels of perceived general health, where those with poorer general health were more likely to consult a medical doctor with PR for the “poor” group compared to the “excellent” group of 1.12 (95%CI: 1.10–1.13).

Prevalence of consultation with a medical doctor was stable over time (PR 1.00 95%CI: 1.00–1.00).

**Physiotherapist** *Predisposing Factors* There was a declining gradient in prevalence across age for consultations with a physiotherapist, with older age groups less likely to consult, with PR for the oldest age group compared to the youngest age group of 0.81 (95%CI: 0.68–0.95). Non-whites were more likely to consult a physiotherapist (PR 1.12 95%CI: 1.02–1.23). There was a gradient across levels of education with the lowest prevalence for those with less than secondary education (PR 0.64 95%CI: 0.60–0.68 relative to post-secondary graduation). *Enabling Factors* There was some variation by province with the lowest prevalence for Newfoundland and Labrador (PR 0.73 95%CI: 0.64–0.83 relative to Ontario) and the highest prevalence for British Columbia (PR 1.22 95%CI: 1.14–1.30 relative to Ontario). There was also a gradient across household income with the lowest income quintile being the least likely to consult a physiotherapist (PR 0.60 95%CI: 0.55–0.67). Based on working status last week, those with a job but absent from work were the most likely to consult a physiotherapist (compared to those working PR 1.44 95%CI: 1.31–1.59). *Need Factors* Smokers (PR 0.79 95%CI: 0.73–0.85 for daily) and inactive persons (PR 0.87 95%CI: 0.82–0.93) were less likely to consult a physiotherapist while regular drinkers (PR 1.16 95%CI: 1.09–1.23) were more likely to consult a physiotherapist. There was a gradient of prevalence across categories of perceived general health with those reporting poor general health with the highest prevalence (PR 2.01 95%CI: 1.78–2.27 compared with excellent general health).

Prevalence of consultation with a physiotherapist increased over time for people with arthritis (PR = 1.06 95%CI: 1.04–1.08).

**Nurse** *Predisposing Factors* Prevalence declined across the first four age groups for consultations with a nurse with older age groups less likely to consult with PR for the second oldest age group 65–79 years compared to 20–29 year olds of 0.49 (95%CI: 0.44–0.54). For the oldest age group 80+ years, the PR compared to 20–29 year olds was 0.65 (95%CI: 0.57–0.75). The following were less likely to consult a nurse: non-whites (PR 0.84 95%CI: 0.75–0.93), immigrants (PR 0.35 95%CI: 0.23–0.54 for immigrants (0–9 years)), those with less education ( < secondary PR 0.77 95%CI: 0.73–0.82 and secondary grad PR 0.80 95%CI: 0.75–0.85). *Enabling Factors* There was some variation by province/territory with the lowest prevalence for Nova Scotia (PR 0.78 95%CI: 0.70–0.87 relative to Ontario) and the highest prevalence for the territories (Yukon, Northwest and Nunavut combined PR 2.82 95%CI: 2.52–3.16 relative to Ontario). Also notable was the higher prevalence of nurse consultations in Quebec compared to Ontario with PR 1.43 (95%CI: 1.35–1.52). Respondents not working were more likely to consult with a nurse than those working during the past week with PRs for those with no job of 1.12 (95%CI: 1.04–1.19), unable to work 0f 1.36 (95%CI: 1.24–1.49) and absent from work of 1.40 (95%CI: 1.25–1.58). *Need Factors* Daily smokers (PR 0.83 95%CI: 0.78–0.88) and regular drinkers (PR 0.93 95%CI: 0.88–0.98) were less likely to consult a nurse while there was less variability in prevalence across activity levels. There was a gradient in prevalence of consulting a nurse across categories of perceived general health, with the highest prevalence ratio for the “poor” group compared to the “excellent” group of PR 2.93 (95% CI 2.60–3.30).

Prevalence of consultation with a nurse showed increases over time (PR 1.09 95%CI: 1.07–1.11).

**Chiropractor** *Predisposing Factors* There was a gradient in PR across age for consultations with a chiropractor with older age groups less likely to consult, with PR for the oldest age group of 0.59 (95%CI: 0.50–0.69). The following were less likely to consult a chiropractor: non-whites (PR 0.88 95%CI: 0.78–0.98), immigrants (PR 0.77 95%CI: 0.71–0.83 for immigrants (10+ years)), and those with less education ( < secondary PR 0.86 95%CI: 0.81–0.91 and secondary grad PR 0.90 95%CI: 0.84–0.97) *Enabling Factors* There was considerable variation by province with lowest prevalence for the eastern provinces with PR (relative to Ontario) of 0.35 for Prince Edward Island (95%CI: 0.27–0.46) and highest prevalence for the western provinces with PR (relative to Ontario) of 1.46 for Alberta (95%CI: 1.36–1.56). Those with lower household income (PR 0.72 95%CI: 0.65–0.80 for lowest quintile), and those with no job (PR 0.81 95%CI: 0.75–0.86) or unable to work (PR 0.77 95%CI: 0.67–0.88) were less likely to consult a chiropractor. *Need Factors* Similar to findings for physiotherapists, smokers (PR 0.76 95%CI: 0.71–0.82 for daily) and inactive persons (PR 0.93 95%CI: 0.87–0.98) were less likely to consult a chiropractor while regular drinkers (PR 1.14 95%CI: 1.07–1.22) were more likely to consult a chiropractor. Compared to those reporting excellent perceived general health, all other categories had increased prevalence of consulting a chiropractor with the highest prevalence ratio for the “fair” group of PR 1.12 95% CI 1.02–1.24).

Prevalence of consultation with a chiropractor showed mild increases over time (PR 1.01 95%CI: 1.00–1.03).

**Psychologist** Overall, confidence intervals for PRs related to psychologists were much wider than for other types of practitioner, reflecting the much lower prevalence of consulting a psychologist. *Predisposing Factors* There was a strong gradient in PR across age for consultations with a psychologist with older age groups less likely to consult, with PR for the oldest age group relative to the youngest group of 0.15 (95%CI: 0.09–0.24). The following were less likely to consult a psychologist: immigrants (PR 0.69 95%CI: 0.58–0.81 for immigrants (0–9 years)), and those with less education ( < secondary PR 0.55 95%CI: 0.47–0.63 and secondary grad PR 0.69 95%CI: 0.58–0.81) *Enabling Factors* There was variation by province with lowest prevalence with PR (relative to Ontario) of 0.80 for Prince Edward Island (95%CI: 0.48–1.34) and highest prevalence with PR (relative to Ontario) of 1.82 for Quebec (95%CI: 1.56–2.13). For work status, those unable to work (PR 1.62 95%CI: 1.32–1.99), absent from work (PR 1.54 95%CI: 1. 25–1.89) or with no job (PR 1.34 95%CI: 1.13–1.58), were more likely to consult a psychologist than those working. *Need Factors* Occasional smokers (PR 1.34 95%CI: 0.97–1.87 compared to non-smokers) and inactive persons (PR 1.09 95%CI: 0.94–1.28) were more likely to consult a psychologist. There is a strong gradient of prevalence across categories of self-rated general health with higher prevalence of consulting a psychologist for all other categories compared to excellent perceived general health (“poor” group PR 3.46 95% CI 2.62–4.55)).

Prevalence of consultation with a psychologist showed increases over time (PR 1.06 95%CI: 1.01–1.12).

#### CCHS 2015–2016: receiving regular healthcare (Table 4)

Across all HCPs, females were more likely to receive regular healthcare from a provider than males with PRs ranging from 1.03 (95%CI: 1.01–1.04) for medical doctor to 1.30 (95%CI: 1.17–1.44) for physiotherapist.

**Medical Doctor** Overall, prevalence ratios across most factors were close to 1.0 indicating small variation across most factors in who reported regular care from a medical doctor over the past 12 months. *Predisposing Factors* There was a gradient across age with older ages more likely to receive regular health care from a medical doctor than younger ages with PR 1.14 (95%CI: 1.09–1.20) for the oldest age group compared with the youngest. *Need Factors* There was a mild gradient across levels of perceived general health where those with poorer general health were more likely to report regular care from a medical doctor with PR for the “poor” group compared to the “excellent” group of 1.04 (95%CI: 1.02–1.07).

**Chiropractor** *Predisposing Factors* There was a gradient in PR across age for regular health care from a chiropractor (similar to *consultations* with a chiropractor 2001–2010) with older age groups less likely to report it, with PR for the oldest age group compared to the youngest age group of 0.63 (95%CI: 0.46–0.88). The following were less likely to report regular health care from a chiropractor: non-whites (PR 0.79 95%CI: 0.66–0.94), immigrants (PR 0.73 95%CI: 0.62–0.87 for immigrants (10+ years)), and those with less education ( < secondary PR 0.87 95%CI: 0.74–1.01 and secondary grad PR 0.92 95%CI: 0.82–1.03). *Enabling Factors* There was considerable variation by province with lowest prevalence for the eastern provinces with PR (relative to Ontario) of 0.26 for New Brunswick (95%CI: 0.19–0.35) and highest prevalence for the western provinces with PR (relative to Ontario) of 1.32 for Alberta (95%CI: 1.15–1.51). Those with lower household income (PR 0.65 95%CI: 0.53–0.79 for lowest quintile compared to highest), and those with no job (PR 0.80 95%CI: 0.71–0.90) were less likely to report regular health care from a chiropractor. *Need Factors* Similar to findings for *consultations* with a chiropractor (2001–2010), smokers (PR 0.59 95%CI: 0.50–0.69 for daily) and inactive persons (PR 0.91 95%CI: 0.81–1.02) were less likely to report while regular drinkers (PR 1.39 95%CI: 1.24–1.57) were more likely to report regular healthcare with a chiropractor. Compared to those reporting excellent perceived general health, all other categories had increased prevalence of regular healthcare with a chiropractor with the highest prevalence ratio for the “fair” group of PR 1.25 95% CI 1.01–1.54).

**Physiotherapist** *Predisposing Factors* For age, the reference group was the youngest age group 20–34, and all other age groups had higher prevalence of regular care with a physiotherapist, with highest prevalence for 35–49 year olds (PR 1.30 95%CI: 0.91–1.87). Non-whites were more likely to report regular care with a physiotherapist (PR 1.20 95%CI: 1.00–1.43). There was a gradient across levels of education, similar to trends with consultations, with the lowest prevalence for those with less than secondary education (PR 0.58 95%CI: 0.47–0.71 relative to post-secondary graduation). *Enabling Factors* There was some variation by province with the lowest prevalence for Newfoundland and Labrador (PR 0.60 95%CI: 0.46–0.79 relative to Ontario) and New Brunswick (PR 0.60 95%CI: 0.47–0.76) and the highest prevalence for Manitoba (PR 1.15 95%CI: 0.92–1.43 relative to Ontario). The gradient across household income was similar to trends for consultations, with the lowest income quintile being the least likely to report regular care from a physiotherapist (PR 0.52 95%CI: 0.43–0.64). Based on working status last week, those with a job but absent from work were the most likely to report regular care from a physiotherapist (compared to those working PR 1.46 95%CI: 1.21–1.76), similar to findings for consultations with a physiotherapist. *Need Factors* Trends were similar to those for consultation with a physiotherapist. Smokers (PR 0.49 95%CI: 0.40–0.59 for daily) and inactive persons (PR 0.87 95%CI: 0.76–1.00) were less likely to report regular care from a physiotherapist while regular drinkers (PR 1.27 95%CI: 1.10–1.47) were more likely to report regular care from a physiotherapist. There was a gradient of prevalence across categories of perceived general health with those reporting poor general health with the highest prevalence (PR 1.88 95%CI: 1.46–2.42 compared with excellent general health).

**Nurse** There is an increasing gradient in prevalence across age groups for regular care from a nurse (unlike the declining prevalence over age groups seen for consultation with a nurse in Table 3) with older age groups more likely to report regular care with PR for the oldest age group 80+ years compared to 20–34 year olds of 1.70 (95%CI: 1.17–2.46). There was some variation by province/territory with the lowest prevalence for British Columbia (PR 0.41 95%CI: 0.32–0.51 relative to Ontario) and the highest prevalence for Prince Edward Island (PR 1.51 95%CI: 1.23–1.86 relative to Ontario). The following were less likely to report regular care from a nurse: non-whites (PR 0.85 95%CI: 0.69–1.04), immigrants 10+ years (PR 0.75 95%CI: 0.63–0.90 compared to non- immigrants), those with less education ( < secondary PR 0.87 95%CI: 0.77–0.98 and secondary grad PR 0.80 95%CI: 0.75–0.85). There was limited variation in prevalence across household income quintiles (PRs ranging from 0.87 to 1.06 across quintiles). Respondents with no job were more likely to consult with a nurse than those working during the past week with PR 1.18 (95%CI: 1.03–1.36. In terms of lifestyle factors, daily smokers (PR 0.87 95%CI: 0.75–1.00) were less likely to report regular care from a nurse than non-smokers while regular drinkers had higher prevalence of regular care from a nurse than non-drinkers (PR 1.09 95%CI: 0.97–1.23).There was a gradient in prevalence of regular care from a nurse across categories of perceived general health, with the highest prevalence ratio for the “poor” group compared to the “excellent” group of PR 2.50 (95% CI 1.96–3.19).

**Psychologist** For age, compared to the reference group age 20–34, prevalence of regular care from a psychologist was higher for 35–49 year olds (PR1.24 95%CI: 0.87–1.79) but lower for all other age groups with a declining gradient with increasing age and PR 0.23 (95%CI: 0.10–0.51) for the oldest age group. There was some variation by province with lowest prevalence with PR (relative to Ontario) of 0.55 for New Brunswick (95%CI: 0.38–0.79) and highest prevalence in Ontario, the reference group. The following were less likely to report regular care from a psychologist: immigrants (PR 0.65 95%CI: 0.47–0.89 for immigrants 10+ years), those with less education ( < secondary PR 0.53 95%CI: 0.40–0.69 and secondary grad PR 0.85 95%CI: 0.67–1.08), and those not in the highest income quintile (PR 0.89 95%CI: 0.66–1.20 for lowest quintile). For work status, those absent from work (PR 2.07 95%CI: 1.58–2.07) or with no job (PR 1.51 95%CI: 1.18–1.94), were more likely to report regular care from a psychologist than those working. In terms of lifestyle factors, occasional smokers (PR 1.48 95%CI: 0.98–2.23 compared to non-smokers) were more likely to report regular care from a psychologist while regular drinkers (PR 0.67 95%CI: 0.52–0.85) and inactive persons (PR 0.90 95%CI: 0.70–1.14) were less likely to report regular care from a psychologist. There is a strong gradient of prevalence across categories of self-rated general health. Compared to those reporting excellent perceived general health, all other categories had increased prevalence of regular care from a psychologist with the highest prevalence ratio for the “poor” group with PR 4.89 95% CI 2.97–8.04).

## Discussion

Among Canadians with arthritis, medical doctors were the most common type of health care provider consulted and providing regular care. From 2015 to 2018, chiropractors were the second most common providing regular care while from 2001 to 2010, physiotherapists were the second most common consulted. Psychologists were the least likely to be either consulted or provide regular care. For all types of practitioners, females were more likely than males to report consultations or regular care in line with other research [36–38]. Associations with the other predisposing, enabling and need related factors varied considerably by practitioner type.

Medical doctors: For Canadians with arthritis, care from medical doctors was the most prevalent for both consultations (2001–2010 – prevalence 92%) and regular care from (2015–2018 – prevalence 91%). The Canada Health Act [24] requires universal coverage of insured health services including physician services enabling all members of the population to access medical doctors via their provincial health insurance plans. This is further borne out in our findings by the flat associations - PRs close to 1 for most factors - indicating limited variation in medical doctor utilization across the factors studied. The strongest associations observed were between age (predisposing) and regular care from a physician (2015–2018) with greater utilization for greater age. The gradient across age for utilization of medical doctors is similar to that seen in the general population of Canada in the National Population Health Survey [39], with increasing utilization of primary care physicians and specialists by age. The age gradient for consultation of medical doctors is also similar to that seen in Canadians with chronic back pain [40] and in Canadians with activity limitations [41]. However, note that the gradient of age we report for regular care with medical doctors is somewhat steeper than that for consultations and than that for regular care among the chronic back pain population [40], perhaps indicating an arthritis specific finding.

Physiotherapists: For consultations (2001–2010), physiotherapists were the second most prevalent care provider reported (14.5%), while for regular care (2015–2018) they were the third most prevalent care provider (9.4%). The strongest associations for physiotherapist utilization were with enabling factor income (greater utilization with higher income and provincial coverage) and need factor perceived general health (greater utilization for those reporting poorer general health). Provincial/territorial differences in physiotherapy utilization to some extent reflect jurisdictional differences in insurance plan coverage for physiotherapy services over time *enabling* access when provinces provided coverage [42–44]. Therefore, the ability to pay for services or the availability of insurance coverage are prominent correlates of physiotherapy utilization in Canadians with arthritis. Jurisdictional differences may also reflect differences in the distribution/availability of physiotherapists geographically as noted by Shah and colleagues [45] who reported “a potential inequality in geographical access”. These access problems could indicate structural determinants of health and function in the Canadian health system. However, our findings indicated an increase in physiotherapy consultations over time between 2001 and 2010, similar to findings reported by others [46].

Nurses: For consultations (2001–2010), nurses were the third most prevalent care provider reported (14.2%), while for regular care (2015–2018) they were the fourth most prevalent care provider (7.5%). The strongest associations with nurse consultations (2001–2010) related to predisposing factors age (lower utilization with increasing age) and immigrant status (less utilization with newer immigrants), enabling factor province (greatest utilization in the Territories and Quebec) and need factor perceived general health with greater utilization for those reporting poorer health. Specifically, geographical variation in utilization of nurses likely reflects lack of availability of medical doctors in some areas of the country. Indeed, Carrière [47] reported that residents of the Territories (Nunavut and the Northwest Territories) and of Quebec were less likely than Canadians overall to consult an MD reflecting the fact that fewer residents of these areas have a regular MD. Conversely, they were more likely to consult a nurse, corresponding to what we report here. In contrast to findings for nurse consultations, regular care from nurses (2015–2018) was highest for the oldest age group. Our findings indicated an increase in nurse consultations over time between 2001 and 2010.

Chiropractors: For consultations (2001–2010), chiropractors were the fourth most prevalent care provider reported (9.6%), while for regular care (2015–2018) they were the second most prevalent care provider (13.1%). Our findings indicated relatively little change in chiropractic consultations over time between 2001 and 2010, similar to findings reported by others [48]. The change in order of utilization from fourth to second likely relates in part to the change in wording of the question from “consultation” to “regular care”, and how respondents interpreted those words. Also, although we did not see increases in utilization of chiropractors from 2001 to 2010 in the Canadian population with arthritis similar to findings reported by others [48], Wong et al. [40] reported an increase in the utilization of chiropractic utilization among Canadians with chronic back problems, suggesting there may be more recognition over time in Canadian society of chiropractic care for dealing with musculoskeletal problems.

The strongest association related to chiropractic utilization was provincial jurisdiction, an enabling factor, with greatest utilization in the western provinces and lowest utilization in Quebec and the eastern Atlantic provinces. This relates to a great extent to differences in coverage in provincial health care plans, but similar geographic variation has been reported in the U.S. where western states show higher utilization [49]. For most provinces, chiropractic services are currently not universally covered by provincial health care insurance plans, but there has been variation across provinces over time in this coverage [43, 50]. Manitoba is currently the only province to include chiropractic care for all (up to seven visits per calendar year) in its provincial plan. However, British Columbia, Alberta, Saskatchewan and Ontario also included some coverage for at least some of the earlier years of our study and some of these provinces continue to provide some coverage through supplementary assistance programs for special needs populations (e.g., elderly, low income). In terms of need factors, the patient profile for chiropractic users is different than that of other practitioners; they are less likely to be smokers, more physically active, regular drinkers, with a flatter gradient over perceived general health, indicating chiropractic patients generally have less need than users of other services in terms of lifestyle factors.

Psychologists: For Canadians with arthritis, care from psychologists was the least prevalent for both consultations (2001–2010 – prevalence 3.0%) and for regular care from (2015–2018 – prevalence 3.9%). While cognitive behavioural therapy is included in the WHO definition of rehabilitation, the low utilization of psychologists here suggests that management of these conditions is still largely within a biomedical model. However, it is possible that other types of providers address psychosocial aspects of living with arthritis such as self-management strategies, advice on activities of daily living and coping. For instance, the GLA:D program (Good Life with osteoArthritis Denmark), a rehabilitation program for OA typically offered in physiotherapy and chiropractic settings, was introduced in Canada toward the end of our study’s observation period and its educational component includes information on self-management and coping [51]. The strongest associations for psychologist utilization were for predisposing factor age, with decreasing utilization with increasing age and need factor perceived general health with greatest utilization for those reporting the poorest health. Our findings indicated an increase in psychologist consultations over time between 2001 and 2010.

### Health systems implications

Canada’s primary healthcare system is in crisis, with more than half unable to see their primary care physician on the same or next day [52]. Unmet health care needs are common among Canadians with chronic conditions, with increased odds among those with arthritis [22]. And yet, our findings indicate the most prevalent healthcare providers seen by Canadians with arthritis were medical doctors at over 90% prevalence, compared to less than 20% prevalence for all other types of provider studied. Geographical variation in utilization of chiropractors and physiotherapists is likely related to differences by province and over time in what provincial health insurance covered while geographical variation in utilization of nurses was likely related to the lack of availability of medical doctors. These findings inform the need to strengthen healthcare delivery for Canadians, by providing better access to providers of rehabilitation interventions.

### Strengths

The CCHS is a population based survey with representation of 98% of community-dwelling Canadians ≥12 years of age [26]. Survey weights are available to generalize findings from the survey and draw inferences to the general population of Canadians with arthritis [26]. The survey covers a long period of 18 years. We considered and investigated associations with health care utilization for a wide variety of predisposing, enabling and need related factors.

### Limitations

Limitations of our study include that there may be measurement error in both the ascertainment of arthritis and in healthcare utilization in that both were based on self-report. However, other studies have used the healthcare utilization questions to describe healthcare utilization in other populations [28–31]. We do not know what type of arthrtitis the respondents had, which may be a factor that influences the type of healthcare utilization they seek, although as noted in the introduction approximately 85% of prevalent arthritis is likely OA. We do not know what the consultations with healthcare practitioners were for nor how many there were (at least for the latest cycles of the first time period) – that is we do not know if they were seeking care for arthritis or if so, from whom. The questions included about health care utilization changed over time from asking about *consultations with* in the earlier cycles to asking about *regular care from* in the later cycles, requiring separate analyses and possibly variations in the associations seen due to wording of the outcome question. It may have been useful to include utilization of pharmacy services also, but this information was not available in the survey. Also, the data are older now, with the most recent available for our study from 2018. However, the age of the data does not impact internal validity of our findings and with the aging of the population and the stability in services compensated by our healthcare systems there is no indication that these trends would change. The CCHS sampling frame only includes individuals living in private dwellings, so results are not be generalizable to other populations such as persons living in institutions or on reserve and other First Nations settlements. Finally, this study is specific to the Canadian population and healthcare system.

## Conclusions

Canadians with arthritis were most likely to see medical doctors. Characteristics of healthcare utilizers varied by provider type. Geographical variation in utilization of chiropractors and physiotherapists likely related to differences in what provincial health insurance covered. Findings inform the need to strengthen healthcare delivery for Canadians, perhaps providing better access to providers of rehabilitation interventions.

## Data Availability

CCHS master files can be accessed at Statistics Canada’s regional offices or regional data centres. Statistics Canada makes available a portion of the CCHS public use microdata file (PUMF), which is designed to preserve statistical analytic capabilities while ensuring respondent confidentiality.

## Appendix 1: STROBE Statement—Checklist of items that should be included in reports of cross-sectional studies

**Table:**

	Item No	Recommendation	
Title and abstract	1	(*a*) Indicate the study’s design with a commonly used term in the title or the abstract	✓ title page
		(*b*) Provide in the abstract an informative and balanced summary of what was done and what was found	✓ page 2
Introduction			
Background/rationale	2	Explain the scientific background and rationale for the investigation being reported	✓ p3
Objectives	3	State specific objectives, including any prespecified hypotheses	✓ p4 top
Methods			
Study design	4	Present key elements of study design early in the paper	✓ 1^st^ paragraph Methods p4
Setting	5	Describe the setting, locations, and relevant dates, including periods of recruitment, exposure, follow-up, and data collection	✓ section starting p4 titled Canadian Community Health Survey
Participants	6	(*a*) Give the eligibility criteria, and the sources and methods of selection of participants	✓ section starting p4 titled Canadian Community Health Survey
Variables	7	Clearly define all outcomes, exposures, predictors, potential confounders, and effect modifiers. Give diagnostic criteria, if applicable	✓ section title Outcome Measures beginning on p5 and section titled Covariates starting on p6
Data sources/measurement	8*	For each variable of interest, give sources of data and details of methods of assessment (measurement). Describe comparability of assessment methods if there is more than one group	✓ for outcomes, questions used provided in section Outcome Measures starting on p5. For covariates, questions used provided in Appendix
Bias	9	Describe any efforts to address potential sources of bias	✓ p4 data source METHODS section Canadian Community Health Survey
Study size	10	Explain how the study size was arrived at	✓ p4 Statistics Canada population-based survey, included all respondents reporting arthritis. Study size described in 1^st^ paragraph of results and in flowcharts. Addressed under section titled Canadian Community Health Survey.
Quantitative variables	11	Explain how quantitative variables were handled in the analyses. If applicable, describe which groupings were chosen and why	✓ section titled Statistical Analysis beginning on p6
Statistical methods	12	(*a*) Describe all statistical methods, including those used to control for confounding	✓ section titled Statistical Analysis beginning on p6
		(*b*) Describe any methods used to examine subgroups and interactions	Not applicable
		(*c*) Explain how missing data were addressed	✓ section titled Statistical Analysis beginning on p6
		(*d*) If applicable, describe analytical methods taking account of sampling strategy	✓ section titled Statistical Analysis beginning on p6 describes use of survey weights
		(*e*) Describe any sensitivity analyses	Not applicable
Results			
Participants	13*	(a) Report numbers of individuals at each stage of study—eg numbers potentially eligible, examined for eligibility, confirmed eligible, included in the study, completing follow-up, and analysed	✓ Flowcharts in Figs. 1 & 2
		(b) Give reasons for non-participation at each stage	✓ Flowcharts in Figs. 1 & 2
		(c) Consider use of a flow diagram	✓ Flowcharts in Figs. 1 & 2
Descriptive data	14*	(a) Give characteristics of study participants (eg demographic, clinical, social) and information on exposures and potential confounders	✓ Tables 1 & 2
		(b) Indicate number of participants with missing data for each variable of interest	✓ Flowcharts in Figs. 1 & 2
Outcome data	15*	Report numbers of outcome events or summary measures	✓ 2nd row of Tables 1 & 2 report prevalences and CI for each HCP type
Main results	16	(*a*) Give unadjusted estimates and, if applicable, confounder-adjusted estimates and their precision (eg, 95% confidence interval). Make clear which confounders were adjusted for and why they were included	✓ Tables 3 & 4
		(*b*) Report category boundaries when continuous variables were categorized	✓ reported in Appendix
		(*c*) If relevant, consider translating estimates of relative risk into absolute risk for a meaningful time period	Not relevant
Other analyses	17	Report other analyses done—eg analyses of subgroups and interactions, and sensitivity analyses	Not applicable
Discussion			
Key results	18	Summarise key results with reference to study objectives	✓ Discussion
Limitations	19	Discuss limitations of the study, taking into account sources of potential bias or imprecision. Discuss both direction and magnitude of any potential bias	✓ Discussion
Interpretation	20	Give a cautious overall interpretation of results considering objectives, limitations, multiplicity of analyses, results from similar studies, and other relevant evidence	✓ Discussion
Generalisability	21	Discuss the generalisability (external validity) of the study results	✓ Discussion – discussed as limitation as this is very specific to Canadian context
Other information			
Funding	22	Give the source of funding and the role of the funders for the present study and, if applicable, for the original study on which the present article is based	✓ p16 section titled Funding

* Give information separately for exposed and unexposed groups

**Note** An Explanation and Elaboration article discusses each checklist item and gives methodological background and published examples of transparent reporting. The STROBE checklist is best used in conjunction with this article (freely available on the Web sites of PLoS Medicine at http://www.plosmedicine.org/, Annals of Internal Medicine at http://www.annals.org/, and Epidemiology at http://www.epidem.com/). Information on the STROBE Initiative is available at http://www.strobe-statement.org

## Appendix 2: Survey questions used for measuring covariates [53–59]

**Table:**

Variable	CCHS Question/Derived variable	Response	Regrouping
Age*	This derived variable has been grouped as a form of disclosure control - Sourced survey question: *“What is your age?”*	0-121 years	1 12–19 2 20–34 3 35–49 4 50–64 5 65–79 6 80+
Sex	*“Is respondent male or female?”*	1 Male 2 Female	/
Province/territory of residence	Administration information based on sample allocation	1 Newfoundland and Labrador 2 Prince Edward Island 3 Nova Scotia 4 New Brunswick 5 Quebec 6 Ontario 7 Manitoba 8 Saskatchewan 9 Alberta 10 British Columbia 11 Yukon/Northwest territories//Nunavut	/
Cultural/racial background* (CCHS 2001–2010)	This derived variable indicates the cultural or racial origin of the respondent - Sourced survey question: *“People living in Canada come from many different cultural and racial backgrounds. Are you … ?”*	1 … White 2 … Chinese 3 … South Asian (e.g., East Indian, Pakistani, Sri Lankan, etc.) 4 … Black 5 … Filipino 6 … Latin American 7 … Southeast Asian (e.g., Cambodian, Indonesian, Laotian, Vietnamese, etc.) 8 … Arab 9 … West Asian (e.g., Afghan, Iranian, etc.) 10 … Japanese 11 … Korean 12 … Aboriginal Peoples of North America (North American Indian, Métis, Inuit/Eskimo) 13 … Other	1 White 2 Non-white (Aboriginal or Other Visible Minority)
Cultural/racial background* (CCHS 2015–2018)	This derived variable indicates the cultural or racial background of the respondent - Sourced survey question: *“you may belong to one or more racial or cultural groups on the following list. Are you … ?”*	1 … White 2 … South Asian (e.g., East Indian, Pakistani, Sri Lankan, etc.) 3 … Chinese 4 … Black 5 … Filipino 6 … Latin American 7 … Arab 8 … Southeast Asian (e.g., Cambodian, Malaysian, Laotian, Vietnamese, etc.) 9 … West Asian (e.g., Afghan, Iranian, etc.) 10 … Korean 11 … Japanese 12 … Other	1 White 2 Non-white (Aboriginal or Other Visible Minority)
	b) *“Are you an Aboriginal person, that is, First Nations, Métis or Inuk (Inuit)?”*	1 Yes 2 No	
Immigrant status*	a) Immigrant flag: this derived variable indicates if the respondent is an immigrant or not.	1 Landed immigrant/non-permanent resident 2 Non-immigrant (Canadian born)	1 Non-immigrant 2 Immigrant who lived in Canada for 0–9 years 3 Immigrant who lived in Canada for 10+ years
	b) Length of time in Canada since immigration: this derived variable indicates the length of time in years the respondent became a landed immigrant.	1 0–9 years 2 ≥10 years	
Education* (CCHS 2001–2010)	This derived variable indicates the highest level of education acquired by the respondent.	1 Less than secondary school graduation 2 Secondary graduation, no post-secondary education 3 Some post-secondary degree/diploma 4 Post-secondary degree/diploma	/
Education* (CCHS2015–2018)	This derived variable indicates the highest level of education attained by the respondent	1 less than secondary graduation 2 Secondary school graduation, no post-secondary education 3 Post-secondary certificate/diploma or university degree	/
Total household income* (CCHS 2001–2010)	This derived variable classifies the total household income into 5 categories based on total household income and the number of people living in the household.	1 Lowest income 2 Lower middle income 3 Middle income 4 Upper middle income 5 Highest income - Population exclusion: residents of territories	/
Total household income* (CCHS 2015–2018)	This derived variable is a distribution of respondents in deciles (ten categories including approximately the same percentage of residents for each province) based on their value for NCDVADR, i.e., the adjusted ratio of their total household income to the low income cut-off corresponding to their household and community size.	1 Decile 1 2 Decile 2 3 Decile 3 … … 9 Decile 9 10 Decile 10 - Population exclusion: residents of territories	For each respondent, a relative measure of their household income to the household incomes of all other respondents: 1 Quintile 1 2 Quintile 2 3 Quintile 3 4 Quintile 4 5 Quintile 5 - Population exclusion: residents of territories
Working status last week*	a) *“Last week, did you work at a job or a business? Please include part-time jobs, seasonal work, contract work, self-employment, baby-sitting and any other paid work, regardless of the number of hours worked.”*	1 Yes 2 No 3 Permanently unable to work - Population exclusions: Age < 15 or > 75	1 Worked at a job or business 2 Had a job but did not work(absent) 3 Permanently unable to work - Population exclusions: Age < 15 or > 75
	b) *“Last week, did you have a job or business from which you were absent?”*	1 Yes 2 No	
Marital status	*“What is [household member name]’s marital status? Is [he/she] … ?”*	1 Married 2 Living common-law 3 Widowed 4 Separated 5 Divorced 6 Single, never married	1 Married 2 Living common-law 3 Widowed/Separated/Divorced 4 Single, never married
Type of smoker	*“At the present time, do you smoke cigarettes daily, occasionally or not at all?”*	1 Daily 2 Occasionally 3 Not at all	/
Type of drinker*	a) *“During the past 12 months, how often did you drink alcoholic beverages?”*	1 Less than once a month 2 Once a month 3 2 to 3 times a month 4 Once a week 5 2 to 3 times a week 6 4 to 6 times a week 7 Every day	1 Regular drinker 2 Occasional drinker 3 Never drank/Did not drink in the last 12 months
	b) *“During the past 12 months, that is, from [date one year ago] to yesterday, have you had a drink of beer, wine, liquor or any other alcoholic beverage?”*	1 Yes 2 No	
Physical activity level* (CCHS 2001–2010)	This derived variable categorizes respondent as being “active”, “moderate”, or “inactive” based on the total daily Energy Expenditure values (kcal/kg/day). The Physical Activity Index follows the same criteria used to categorize individuals in the Ontario Health Survey (OHS) and in the Campbell’s Survey on Well Being.	Energy Expenditure is calculated using the frequency and duration per session of the physical activity as well as the MET value of the activity. The MET is a value of metabolic energy cost expressed as a multiple of the resting metabolic rate. For example, an activity of 4 METS requires four times the amount of energy as compared to when the body is at rest. 1 Active 2 Moderate active 3 Inactive	/
Physical activity level* (CCHS 2015–2018)	This derived variable indicates whether a respondent is physically active according to the recommended level from Canadian Physical Activity Guidelines (CPAG): at least 150 minutes of moderate- to vigorous-intensity aerobic physical activity per week, in bouts of 10 minutes or more.	Based on the total number of minutes a respondent engaged in active transportation and moderate to vigorous recreational and other physical activities: 1 Physically active at/above recommended level from CPAG 2 Physically active below recommended level from CPAG 3 No physical activity minutes reported	/
Body mass index*	A derived variable assigns adult respondents aged 18 and over (except pregnant women) to one of the following categories, according to their Body Mass Index (BMI) and based on classification system recommended by Health Canada and the World Health Organization	1 Underweight 2 Normal/Acceptable weight 3 Overweight 4 Obese class I, II, or III (available for CCHS 2003–2018) - Population exclusion: age < 18 or pregnant women	1 Underweight 2 Normal/Acceptable weight 3 Overweight (including Obese class I, II, or III) - Population exclusion: age < 18 or pregnant women
Self-perceived general health	*“In general, would you say your health is … ?”*	1 Excellent 2 Very good 3 Good 4 Fair 5 Poor	/

* Derived variables are produced by Statistics Canada for Public Use Microdata File (PUMF) of Canadian Community Health Survey, accessed via Ontario Data Documentation, Extraction Service and Infrastructure Initiative

## Abbreviations

AS: Ankylosing Spondylitis
CCHS: Canadian Community Health Survey
CCRF: Canadian Chiropractic Research Foundation
CDC: Centers for Disease Control
CI: Confidence Interval
CIHI: Canadian Institute for Health Information
HCP: Health Care Practitioner
NSAIDs: Non-Steroidal AntiInflammatories
ODESI: Ontario Data Documentation, Extraction Service and Infrastructure Initiative
PA: Psoriatic Arthritis
PR: Prevalence Ratio
PUMF: Public Use Microdata File
RA: Rheumatoid Arthritis
STROBE: Strengthening the Reporting of Observational Studies in Epidemiology
TENS: Transcutaneous Electrical Nerve Stimulation
US: United States
WHO: World Health Organization
